# Highly Improved Sensitivity of FET Sensors Based on a Pulsed Temperature Profile for Lead Ion Detection

**DOI:** 10.1021/acs.analchem.5c07438

**Published:** 2026-04-07

**Authors:** Guan-Cheng Zeng, Yi-Te She, Ching-Yao Lan, Yi-Fang Wu, Chia-Kai Lin, Hsuan-Wei Huang, Jung-Chih Chen, Sheng-Chun Hung, Guo-Chun Dong, Yu-Lin Wang

**Affiliations:** † Institute of Nanoengineering and Microsystems, 34881 National Tsing Hua University, Hsinchu 300044, Taiwan; ‡ Department of Power Mechanical Engineering, National Tsing Hua University, Hsinchu 300044, Taiwan; § Department of Medical Laboratory Science and Biotechnology, Central Taiwan University of Science and Technology, Taichung 406053, Taiwan; ∥ Department of Electrical Engineering, 34902 Feng Chia University, Taichung 407102, Taiwan; ⊥ Institute of Biomedical Engineering, 34914 National Yang Ming Chiao Tung University, Hsinchu 300093, Taiwan; # Institute of Biomedical Engineering and Nanomedicine, 50115 National Health Research Institutes, Miaoli 350401, Taiwan; ¶ College of Semiconductor Research, National Tsing Hua University, Hsinchu 300044, Taiwan

## Abstract

This study presents the development of a lead-based molecular probe integrated with an extended-gate field-effect transistor (EGFET) for the highly sensitive detection of lead ions (Pb^2+^). The molecular probe was carefully selected and engineered to exhibit a strong binding affinity for Pb^2+^ while maintaining both thermodynamic stability and structural robustness. To enhance sensing performance, we employed a pulsed temperature profile strategy that accelerates molecular interactions and overcome energy barriers, followed by a stabilization step to preserve the integrity of the formed complexes. Through this approach, the sensor achieved an ultralow detection limit of 1 pM, highlighting the crucial role of molecular probe structural stability and thermodynamic properties in improving overall sensing efficiency. DNA linkers functionalized with reactive groups and fluorescent labels were employed to enable real-time monitoring of surface modification and facilitate efficient molecular immobilization, thereby enhancing the sensor’s versatility. Moreover, constructing a monolayer sensing interface allowed direct regulation of the electric double layer (EDL), resulting in rapid signal responses. Selectivity tests confirmed the sensor’s preferential interaction with Pb^2+^ ions. Overall, this study developed an innovative sensing platform that achieves ultralow detection limits and high selectivity for Pb^2+^. The platform is highly versatile and can be extended to EGFET-based sensing of a wide range of molecules.

## Graphical abstract

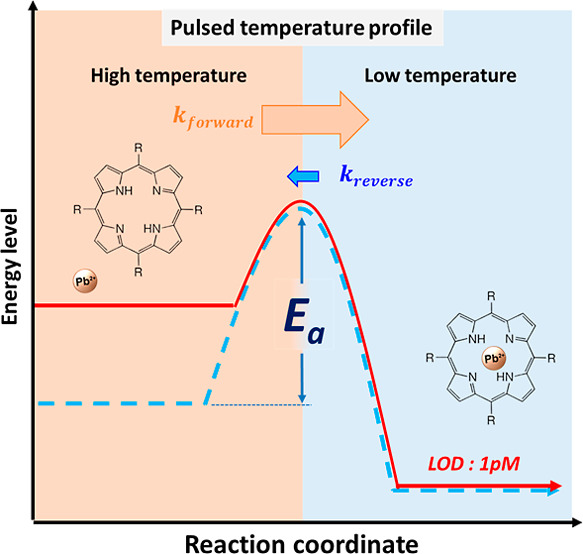

## 1 Introduction

Heavy metals such as lead (Pb), cadmium (Cd), mercury (Hg), and arsenic (As) pose significant threats to both human health and the environment. These contaminants primarily originate from mining, metal smelting, agricultural activities, and improper waste disposal.^1,2^ Among these metals, lead is considered one of the most representative pollutants due to its widespread presence and strong bioaccumulation potential. Extensive studies have demonstrated that lead exposure damages the nervous and hematopoietic systems, impairs renal and cardiovascular functions, and exhibits both genotoxic and carcinogenic properties, with risks present even at trace levels.^3–5^ More recent findings further indicate that lead can trigger multiple inflammatory pathways, affecting the respiratory, neurological, digestive, cardiovascular, and renal systems. Alarmingly, an estimated 14–17% of global farmland (approximately 242 million hectares) is contaminated with toxic heavy metals,^6^ including lead, posing significant challenges to food security, agricultural productivity, and public health—particularly in Asia, Africa, and the Middle East. To effectively monitor environmental heavy metal pollution, the choice of analytical techniques is crucial. Energy-dispersive X-ray fluorescence (ED-XRF) is a fast and reliable analytical method widely applied for field-based assessment of contaminated environments. It enables simultaneous multielement detection, with typical sensitivity levels ranging from 0.01 to 1 μg/g.^7,8^ In comparison, atomic absorption spectroscopy (AAS) can achieve sensitivities down to the ppb level,^9^ while inductively coupled plasma mass spectrometry (ICP-MS) attains ppt detection limits,^10^ offering the highest sensitivity and the capability for simultaneous multielement analysis. This makes ICP-MS the gold standard in laboratory settings.^11^ Despite their powerful analytical capabilities, these techniques are costly and require specialized operators, limiting their applicability for rapid, on-site monitoring. Therefore, there is a pressing need for novel detection methods that combine high sensitivity with portability and cost-effectiveness.

Considerable efforts have focused on developing lead sensors that exploit the specific binding between aptamers and Pb^2+^ ions. Among different detection technologies, electrochemical sensors and FET-based systems have shown exceptional performance, reaching detection limits from below the sub-pM level up to the sub-μM range.^12–18^ Nonetheless, the detection limits reported across different studies show substantial variation, mainly attributed to disparities in charge-transfer efficiency. This variation often arises from the distinct electrical and structural characteristics of nanomaterial-coated electrodes employed in voltammetric sensing systems. In aptamer-based designs for Pb^2+^ recognition, the aptamer typically adopts a G-quadruplex (G4) structure, formed by four guanine-rich sequences stacked via Hoogsteen hydrogen bonding.^19^ Pb^2+^ ions stabilize the G4 structure by inserting into its central channel.^20^ Nonetheless, in FETs or other surface-immobilized sensors, excessively high immobilization density of G4 aptamers can hinder Pb^2+^ access to binding sites. Overcrowding induces steric hindrance,^21^ reducing binding efficiency and thereby compromising sensor sensitivity. To mitigate this effect, flexible linkers or spacers are commonly introduced between aptamers to ensure full exposure of the G4 structures, enhancing Pb^2+^ binding efficiency and sensor performance.^22^ In addition, the design of aptamers requires careful optimization of their secondary structure, melting temperature, and compatibility with the sensing environment. Ensuring that the operating temperature remains below the aptamer’s melting temperature is essential to preserve its conformation and enable reliable detection at trace concentrations.^23^ These constraints pose notable challenges for aptamer-based Pb^2+^ detection, particularly in complex real-world sample matrices.^24,25^

Molecular probes represent a promising approach for ion recognition due to their ability to reversibly bind target ions.^26,27^ Their design typically incorporates structural motifs, including crown ethers, porphyrins, and calixarenes, which provide well-defined binding cavities for selective ion capture.^28–38^ The geometry and local coordination structure around the metal ion, especially the identity and quantity of coordinating donor atoms, play a crucial role in determining the catalytic and binding properties of molecular probe–metal complexes.^39^ Such structural characteristics critically govern their ion selectivity and binding affinity, rendering them promising materials for targeted ion recognition and sensing applications.^40^ Molecular probes have already been widely employed in ion-selective membranes (ISMs),^41,42^ including ion-selective electrodes (ISEs),^43–48^ AlGaN/GaN high electron mobility transistors (HEMTs),^49^ and field-effect transistors (FETs)^50–52^ for the detection of lead ions. Lee et al.^53^ developed a poly­(vinyl chloride) (PVC) membrane electrode incorporating meso-tetrakis­(2-hydroxy-1-naphthyl)­porphyrin, achieving a detection limit of 3.5 × 10^–6^ M. Chen et al.^49^ further demonstrated an ion-selective high-electron-mobility transistor (ISHEMT) sensor, providing an ultralow detection limit of 10^–10^ M. More recently, Wang et al.^52^ developed an impedance-modulated field-effect transistor (ISM-FET) sensor integrated with a portable measurement unit, also achieving a remarkably low detection limit of 10^–10^ M for Pb^2+^. However, the fabrication of ion-selective membranes (ISMs) typically involves spin-coating polymer films onto electrode surfaces. This method often produces relatively thick coatings, which slow down ion diffusion and result in baseline stabilization times exceeding 15 min—limiting their suitability for rapid analyses. In aqueous environments, interfacial potentials arising at the membrane–solution interface^54–56^ further affect ion transport,^57^ reducing sensing efficiency. Additionally, ISM-coated sensors generally require pretreatment, such as preconditioning with concentrated ion solutions, to establish equilibrium within the membrane matrix,^51,52,58,59^ thereby increasing operational complexity. These limitations underscore the need for sensor designs that minimize membrane thickness, mitigate interfacial effects, and enhance durability against contamination to ensure reliable field applications.

In this study, we developed a sensing platform based on a monolayer of functional molecules, which significantly enhances reaction kinetics. DNA was employed as a linker to overcome the slow diffusion associated with thick films, while the molecular probe was immobilized via robust amide bonds. Additionally, fluorescently labeled DNA enabled monitoring of surface functionalization. According to M. Goldberg et al.,^60^ the formation of amide bonds can quench the fluorescence of attached dyes, allowing the density of the molecular probe to be quantitatively measured through changes in fluorescence intensity. By tracking fluorescence signals at each stage of surface modification, precise control over sensor fabrication quality was achieved.

4,4′,4″,4‴-(Porphine-5,10,15,20-tetrayl)­tetrakis­(benzoic acid) has been employed as a molecular probe^61–66^ for lead ions. The interaction of lead ions with porphyrin derivatives has been widely investigated, with detailed characterization performed using methods including nuclear magnetic resonance (NMR)^61,62^ and infrared spectroscopy (IR).^64,65^ Gibbs free energy analysis shows that the formation of the complex occurs spontaneously, as reflected by the negative value of Δ*G*. Nevertheless, numerous studies have revealed that the interactions between molecular probes and heavy metal ions encounter an activation energy barrier,^67,68^ a factor rarely addressed in existing sensor literature. Given the critical role of reaction kinetics in sensor performance, we introduced a pulsed temperature profile strategy to enhance sensitivity and investigate its underlying mechanism. Additionally, the hydration shell of lead ions influences their adsorption on molecular probe, prompting further investigation into how salt concentration modulates this effect on sensor signals.

To effectively amplify Pb^2+^-induced surface potential changes, a high-transconductance EGFET configuration was employed. This setup preserves the intrinsic characteristics of the FET, as the sensing solution does not directly contact the transistor materials, thereby providing high transconductance precision, cost-effectiveness, and user-friendliness. Importantly, the double-layer effect increases carrier density, significantly enhancing sensitivity while enabling ultralow power operation—features critical for detecting trace levels of lead ions. By combining a monolayer architecture, temperature modulation, and the EGFET design, this platform facilitates rapid signal transduction, precise monitoring of surface functionalization via fluorescence, and tunable sensor performance through salt concentration control. The developed sensing system achieved an ultralow detection limit of 1 pM for Pb^2+^, highlighting its strong promise for use in biosensing applications.

## 2 Materials and Experimental

### 2.1 Experimental Materials and Chemical Reagents

All reagents used in this study were of analytical grade and were applied without further purification unless otherwise specified. Mercury nitrate (Hg­(NO_3_)_2_), lead nitrate (Pb­(NO_3_)_2_), cadmium nitrate (Cd­(NO_3_)_2_), zinc nitrate hexahydrate (Zn­(NO_3_)_2_·6H_2_O), copper­(II) sulfate (CuSO_4_), nickel­(II) sulfate (NiSO_4_), iron­(III) chloride hexahydrate (FeCl_3_·6H_2_O), magnesium chloride (MgCl_2_), calcium chloride (CaCl_2_), potassium chloride (KCl), potassium chromate (K_2_CrO_4_), tin­(II) chloride dehydrate (SnCl_2_·2H_2_O), thallium­(I) nitrate (TlNO_3_) and sodium meta-arsenite (NaAsO_2_) were supplied by Sigma-Aldrich. To maintain consistent ionic strength during testing, all solutions were prepared using the same buffer base. Additional chemicals, including phosphate-buffered saline (10X PBS), 2-(*N*-morpholino)­ethanesulfonic acid (MES), 1-ethyl-3-(3-(dimethylamino)­propyl)­carbodiimide (EDC), and *N*-hydroxysulfosuccinimide (sulfo-NHS), were also sourced from Sigma-Aldrich.

Deionized water was used to dilute PBS to the desired concentrations (1X, 0.1X, 0.02X, 0.01X, and 0.001X), which were employed interchangeably as assay and rinsing buffers throughout all experimental procedures. For surface activation via EDC/sulfo-NHS chemistry, a freshly prepared 0.1 M MES buffer containing 0.5 M NaCl was utilized. To ensure reproducibility and minimize contamination, all solutions were freshly prepared before each experimental run.

### 2.2 Surface Modification

Before surface modification, the sensor chips were first exposed to an 18 W oxygen plasma for 3 min to enhance surface cleanliness and reactivity. They were then immersed in a 4:10 diluted hydrochloric acid solution and ultrasonically cleaned for 10 min to remove any remaining oxide layers, followed by thorough rinsing with deionized water. A DNA working solution at a concentration of 5 μM was prepared by diluting a 100 μM stock solution (1:19 v/v) with DI water. This solution was drop-cast onto the gold electrode region. The DNA linker (5′-SH-AAAAAAAAAAAAAAAAAAAAAAAAAAAATA-NH_2_-3′, dT-FAM, 30-mer), synthesized by Tools Biotechnology Co., Ltd., contained a thiol group at the 5′ terminus, an amine group at the 3′ terminus, and a fluorescein (FAM) fluorophore attached to a thymine base. The chips were incubated for 24 h at 24 °C to promote covalent immobilization. After incubation, unbound DNA molecules were washed away using 1X PBS preheated to 95 °C. Fluorescent images were captured with a LEICA DM2500 LED microscope, and the signal intensity was quantified using ImageJ software.

For molecular probe coupling, a 10^–5^ M solution of 4,4′,4″,4‴-(porphine-5,10,15,20-tetrayl)­tetrakis­(benzoic acid) was freshly prepared in 0.1 M MES buffer containing 0.5 M NaCl. To activate the carboxyl groups, 0.4 mg of EDC (final concentration 2 mM) and 1.1 mg of sulfo-NHS (final concentration 5 mM) were added and allowed to react for 15 min at room temperature. The activated solution was then applied to the DNA-functionalized electrodes and incubated for 2 h to facilitate amide bond formation. Following conjugation, the chips were rinsed multiple times with 1X PBS to remove any residual reactants. Fluorescence microscopy and ImageJ analysis were subsequently performed to confirm the successful attachment of the molecular probe.

### 2.3 Bio-EGFET Sensing Platform

The fabricated sensor chip was incorporated into a compact readout platform that allowed continuous signal acquisition through a customized software interface running on a laptop. As depicted in Figure 1A, the sensing window, measuring 450 × 450 μm^2^, was defined using a polymeric membrane to precisely confine the reaction area. The underlying printed circuit board (PCB) contained eight individual sensing units, each featuring a pair of gold electrodes spaced 180 μm apart. Every electrode pair was connected to an n-channel enhancement-mode MOSFET (VN10LP). Although depletion-mode FETs are also suitable for BioFET systems, enhancement-mode devices were selected for this design due to their higher transconductance (*g*_m_), which provides more efficient signal amplification. Signal acquisition was carried out by applying a periodic square-wave voltage to the gate with a 2 ms on/2 ms off pulse cycle and a 100 ms idle interval, as illustrated in Figure 1B. Upon immersion in the electrolyte, an electrical double layer (EDL) formed at the electrode–solution interface, influencing the transistor response through capacitive coupling. The transistor characteristics were analyzed using a semiconductor parameter analyzer (Agilent/Keysight B1500A). The output profiles in Figure 1C show the drain current as a function of drain voltage at various gate potentials, while the transfer curve in Figure 1D was measured at a fixed drain bias of 3.5 V. The maximum transconductance, 128.3 mS, occurred near *V*_g_ = 3 V, corresponding to the highest sensitivity to gate potential perturbations. The sensing operation is governed by EDL modulation at the gate-electrolyte boundary—binding events alter the interfacial potential, and these small potential shifts are transduced and amplified by the BioFET, enabling detection of subtle analyte concentration variations as represented by eq 1.^23^

1 $$g_{m=}{\left[\partial I_{d}/\partial v_{g}\right]}_{v}d$$

**Figure 1:**
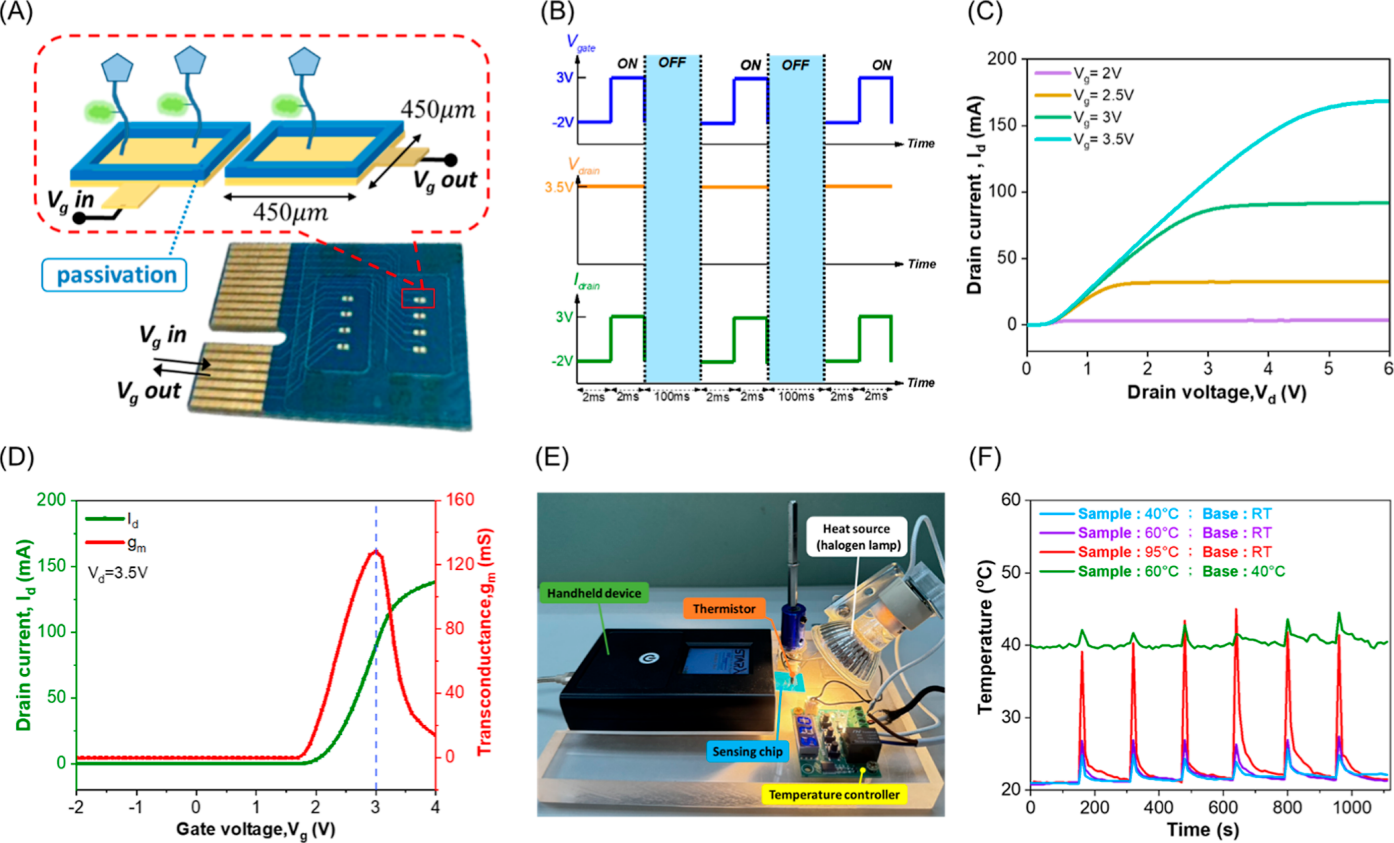
Schematic illustration and key parameters of the bio-EGFET sensing platform and the temperature control module. (A) The EGFET array consists of eight sensors arranged in a 2 × 4 layout, each featuring gold electrodes measuring 450 μm × 450 μm. (B) A square-wave voltage with 2 ms ON/OFF pulses and 100 ms intervals was applied to the gate and drain. (C) The drain voltage was swept from 0 to 6 V at a constant gate voltage to record the *I*_d_–*V*_d_ characteristics. (D) The gate voltage was scanned from −2 to 4 V at a drain bias of 3.5 V to obtain the transfer and transconductance curves. (E) The temperature regulation subsystem operates through a closed-loop feedback mechanism, where the thermistor signal is processed by a control circuit to modulate the halogen lamp intensity, ensuring accurate thermal stability. (F) Temperature response curves were recorded under three test conditions: when samples preheated to 40 and 60 °C were introduced into a solution at room temperature (21 °C), and when a 60 °C sample was added to a 40 °C baseline solution. The transient spikes observed in each curve correspond to the injection of 10% (v/v) of the heated sample into the sensing chamber.

Equations 2 and 3 illustrate the operating principles of the BioFET system, which is governed by the EDL. The EDL can be conceptually divided into two primary regions: the compact Stern layer and the diffuse layer. This theoretical framework follows the classical Gouy–Chapman–Stern model, where ε_0_ is the vacuum permittivity; ε*_r_* represents the dielectric constant of the Stern layer; *l*_st_ denotes its thickness; A corresponds to the electrode surface area; ε_*r*_ is the dielectric constant of the diffuse layer; and *l*_diff_ approximates the Debye screening length. The formation of the EDL results in an effective interfacial capacitance, with the overall EDL capacitance expressed as a series combination of the Stern layer capacitance (*C*_St_) and the diffuse layer capacitance (*C*_diff_), as shown in eq 4.^69^

2 $$C_{St}=\varepsilon_{0}\varepsilon_{r,st}A/l_{st}$$

3 $$C_{diff}=\varepsilon_{0}\varepsilon_{r}A/l_{diff}$$

4 $$1/C_{EDL}=1/C_{st}+1/C_{diff}$$

The surface potential (φ_0_) influences the capacitance of the electrical double layer (*C*_EDL_), and their relationship is described by eq 5.^70^ Additionally, the Grahame equation^71^ (eq 6) relates the surface potential to the surface charge density, where σ_0_ represents the surface charge, σ_EDL_ is the charge within the EDL, *c*_0_ is the bulk electrolyte concentration, ε denotes the relative permittivity of the buffer solution, *k*_B_ is Boltzmann’s constant, *T* is the absolute temperature, *e* is the elementary charge, and φ_St_ corresponds to the potential at the Stern plane. When Applying a gate voltage to the extended-gate electrode induces a potential drop between the electrode and the solution, resulting in the formation of an EDL at the metal–electrolyte interface. This applied gate voltage is then transmitted to the MOSFET gate terminal, modulating the channel conductance of the FET.^72^ The corresponding model is described in eq 7.^23^

5 $$\sigma_{EDL}=-\sigma_{0}=-C_{EDL}\cdot \varphi_{0}$$

6 $$\sigma_{EDL}=\sqrt{8{\varepsilon \varepsilon }_{0}k_{B}T}\cdot \sin h\left(e\varphi_{St}/{2k}_{B}T\right)$$

7 $$V_{g}+{\Delta V}_{g}=V_{g,ext}$$

the Δ*V*_g_ represents the potential difference between the extended-gate electrode and the target ions, while *V*_g,ext_ denotes the residual gate voltage. The EDL-FET can be modeled using an equivalent RC circuit, as described in previous studies.^73–75^ The capacitance of the EDL plays a critical role because it directly influences the voltage applied to the MOSFET gate terminal. Interactions between the EDL and target analytes induce changes in the EDL capacitance, which in turn modulate the gate voltage’s effect on the FET channel. This modulation alters the channel conductivity, producing measurable variations in the drain current. By monitoring these current changes, the sensor can detect and quantify specific ions or molecules in the solution, enabling sensitive and precise measurements based on the capacitance-dependent response of the EDL.

### 2.4 Temperature Control Assembly

A poly­(methyl methacrylate) (PMMA) base plate measuring 210 mm × 150 mm × 20 mm was fabricated using computer numerical control (CNC) machining to provide mechanical support for the thermal regulation system. The assembly included a temperature controller (model XH-W1209-5 V-BLUE, 48.5 mm × 40 mm), a 10 kΩ NTC thermistor (103AT-4 Shape 2), and a 110 V, 50 W GU10 halogen lamp. The halogen lamp was positioned to directly illuminate the sensing region, while the thermistor continuously monitored the solution temperature and transmitted the signal to the controller. Through a real-time feedback mechanism, the system adjusted the lamp intensity to maintain the solution temperature within ±0.5 °C, as shown in Figure 1E.

The temperature variations recorded under different testing scenarios are shown in Figure 1F. In the case represented by the blue trace (sample at 40 °C; base at room temperature), the system started at 21 °C. The injection of a preheated sample (10% of the total volume) caused a rapid increase to approximately 25 °C, followed by a swift return to the original temperature. Similar responses were observed when samples at 60 °C (purple) and 95 °C (red) were introduced, producing transient peaks before returning to equilibrium. In the scenario where a 60 °C sample was added to a 40 °C baseline solution, the resulting curve confirmed the controller’s ability to maintain thermal stability under dynamic temperature changes.

### 2.5 Sensing Procedure and Analyte Introduction

As illustrated in Figure 1A, a patterned waterproof tape was applied to the chip surface to physically separate the upper and lower electrode arrays while simultaneously defining the sensing area. This tape-defined configuration confined the sensing solution to the electrode region, enabling sensing measurements without the need for microfluidic channels. Prior to analyte introduction, 50 μL of 0.02X PBS was added to each sensing area to stabilize the electrical baseline. After baseline stabilization, Pb^2+^ solutions of varying concentrations were sequentially introduced by incremental volume additions using a micropipette, allowing precise control of the effective analyte concentration within the sensing chamber. Specifically, 5.55 μL of a 10^–10^ M Pb^2+^ solution was first added to the sensing chamber, resulting in a final Pb^2+^ concentration of 10^–11^ M. Subsequently, Pb^2+^ solutions of increasing concentrations were introduced in the same manner: 6.17 μL of 10^–9^ M, 6.85 μL of 10^–8^ M, 7.62 μL of 10^–7^ M, 8.40 μL of 10^–6^ M, and 9.40 μL of 10^–5^ M, respectively. After each addition, the solution was gently mixed by pipetting to ensure a homogeneous distribution of the analyte within the sensing area. The electrical response of the sensor was recorded after signal stabilization at each concentration, allowing for the evaluation of sensing performance across a broad dynamic concentration range.

### 2.6 Attenuated Total Reflection Fourier Transform Infrared Spectroscopy

The sequential surface modification of the sensor was confirmed via ATR-FTIR spectroscopy using a Bruker Tensor II instrument fitted with a Platinum ATR unit containing a diamond crystal. Before measurements, samples were rinsed with deionized water and gently nitrogen-dried to remove impurities and suppress background noise. Spectral data were obtained from 4000 to 400 cm^–1^ at 4 cm^–1^ resolution, with each spectrum representing the mean of 32 scans for improved signal-to-noise performance.

### 2.7 Molecular Dynamics and Free Energy Calculation Parameters

ORCA 5.0 program package^76^ was used to investigate the interaction between Pb^2+^ and 4,4′,4″,4‴-(porphine-5,10,15,20-tetrayl)­tetrakis­(benzoic acid) (molecular probe). The B3LYP exchange–correlation functional was employed in combination with the def2-SVP basis set for all atoms. Grimme’s D3 dispersion correction with Becke–Johnson damping (D3BJ) was included to account for noncovalent interactions. Solvent effects were incorporated using the SMD (solvation model based on density) implicit solvation model, with water as the solvent. For calculations at 298 K, the default dielectric constant of water (ε = 78.36) was used, while calculations at 333 K employed a temperature-corrected dielectric constant (ε = 66.77) to account for the temperature dependence of solvent properties. The free energy profile was constructed by performing a relaxed potential energy surface scan along the Pb–N_4_ distance coordinate, where N_4_ represents the geometric center of the four pyrrolic nitrogen atoms in the porphyrin core. The Pb–N_4_ distance was varied from 0 to 40 Å to capture the entire binding process, from the separated state to the fully coordinated complex. The molecular probe ligand was modeled in its fully deprotonated form (net charge −4), with all four carboxylic acid groups ionized, resulting in a total system charge of −2 for the molecular probe-Pb^2+^ complex. Electronic energies obtained from DFT calculations were converted to Gibbs free energies by incorporating translational entropy corrections estimated using the Sackur–Tetrode equation for the ion separation process.

### 2.8 Real Sample Pretreatment

In this study, river water and grape seed extract were selected as representative real-world samples. River water was collected from the Touqian River near Hsinchu City, transferred into 50 mL centrifuge tubes, and subsequently filtered through 25 mm, 0.45 μm PTFE syringe filters (Biofil, JET BIOFIL, China) for pretreatment. The grape seed extract used in this study was obtained from Moi International Enterprise Co., Ltd. and supplied as a standardized commercial product that had undergone purification prior to distribution. The extract was first diluted with deionized water at a 1:1 ratio. The oil in the sample was removed by adsorption using oil-absorbing cotton (SCiKET, SABZT0001-0007BK, Taiwan) overnight, followed by filtration through a 0.45 μm PTFE membrane. To adjust the conductivity, 5 mL of the filtrate was mixed with 446 μL of 10X PBS, resulting in a final conductivity of 354 μS/cm.

### 2.9 SLRS-6 Pretreatment and Measurement Procedure

A certified reference material (CRM), SLRS-6 (National Research Council Canada), with an initial pH of 2.45, was used in this study. The pH was first adjusted to 6.5 by adding 57 μL of 1 M NaOH to 5 mL of SLRS-6. The conductivity of the adjusted solution was measured at 1.22 mS/cm. Subsequently, the solution was diluted 4-fold with DI water, resulting in a conductivity of 364 μS/cm. Finally, one-tenth of the prepared sample was introduced into the sensor for current signal measurement.

## 3 Results and Discussion

### 3.1 Surface Modification

In this work, a detection platform was developed by leveraging the functional versatility of DNA and fluorescent labeling to enable controlled surface modification and monitoring. The thiol group modified at the 5′ end of the DNA forms a stable Au–S bond with the gold electrode, thereby firmly anchoring the strand to the electrode surface. The 3′ amino group reacted with the carboxyl groups of the molecular probe via EDC/sulfo-NHS chemistry, covalently immobilizing the sieve via amide bond formation. A thymine-linked FAM fluorophore enabled observation of surface modification via fluorescence signal changes.

## 4 Results of Molecular Dynamics Simulations

Figure 2A illustrates the sequential surface modification strategy. Initially, the DNA linker was anchored onto the gold electrode, followed by the covalent attachment of the molecular probe via EDC/sulfo–NHS–mediated cross-linking, completing the functionalization process. Figure 2B presents fluorescence monitoring at each step: immobilization of the DNA linker increased the signal from 7.19 to 45.5, while subsequent conjugation with the molecular probe reduced it to 9.85, approaching the baseline observed for bare gold. Previous studies have reported that amide bond formation can quench red-shifted fluorophores.^60^ In this system, the molecular probe was covalently linked to the DNA’s terminal amine through amide bonds formed with its carboxyl groups, consistent with structures described in earlier research.^60^ This quenching effect is likely related to a photoinduced electron transfer (PET) mechanism, although the exact pathway remains to be elucidated. Overall, these observations highlight both the molecular processes underlying surface functionalization and the utility of FAM labeling for real-time monitoring of modification efficiency, thereby supporting the evaluation of sensor performance.

**Figure 2:**
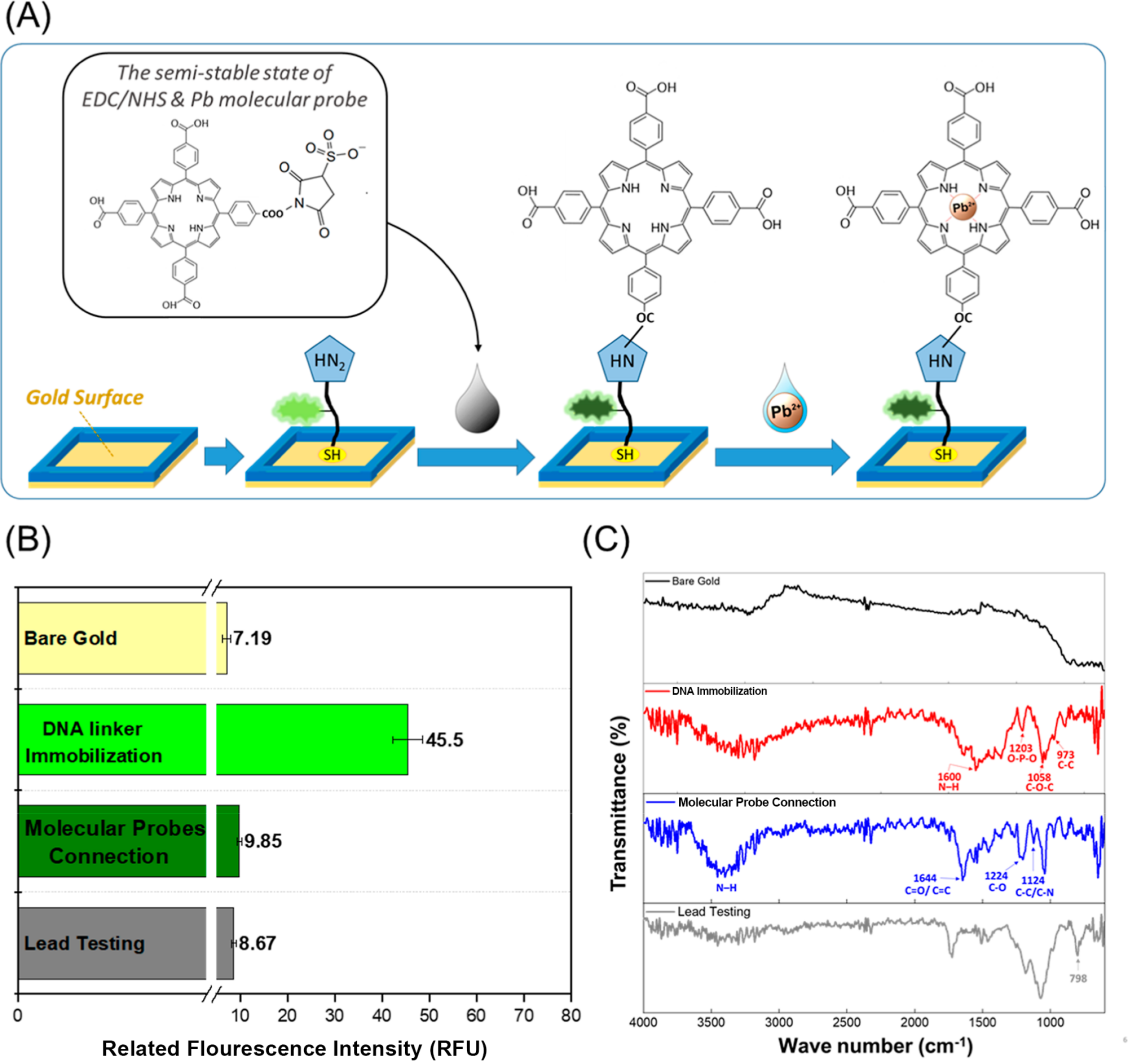
Surface modification characterization results. (A) Diagram illustrating the stepwise surface modification of the gold electrode. DNA linkers were first immobilized on the surface, followed by the covalent attachment of molecular probes via EDC/sulfo-NHS coupling chemistry. (B) Comparison of fluorescence signals corresponding to different surface states: unmodified gold, after DNA linker attachment, after molecular probe functionalization, and following lead ion binding. (C) ATR-FTIR spectra revealed characteristic vibrational changes at each stage of surface modification, including the bare gold surface, the DNA-functionalized layer, the molecular probe-conjugated surface, and following interaction with lead ions.

Figure 2C presents the FTIR transmittance spectra at each stage of surface functionalization. The red curve represents DNA immobilized on the substrate. The feature at 973 cm^–1^ is attributed to the stretching vibration of the ether backbone (ν­(C–C)), while the band at 1058 cm^–1^ corresponds to the C–O–C stretching vibration (ν­(C–O–C)).^77–80^ The signal at 1203 cm^–1^ arises from vibrations of the O–P–O moieties in the DNA phosphate backbone,^80^ and the band near 1600 cm^–1^ is associated with N–H bending of the amino groups attached to the DNA.^81^ The blue curve represents the spectrum following the covalent attachment of the molecular probe. The band at 1124 cm^–1^ is assigned to the C–C and C–N skeletal stretching vibrations of the molecular probe core (ring skeletal vibration),^82^ while the feature at 1224 cm^–1^ corresponds to C–O and carboxyl-related fingerprint modes.^83^ The peak observed at 1644 cm^–1^ is attributed to a combination of the C=O stretching of the carboxylic acid groups (–COOH) on the molecular probe periphery and the C=C stretching vibrations of the molecular probe, indicating both the presence of carboxyl functional groups and the integrity of the conjugated molecular ring system.^84^ The broad band between 3310 and 3400 cm^–1^ arises from the N–H stretching vibrations of the inner pyrrolic NH of the molecular probe.^83^ Upon Pb^2+^ binding (gray curve), the N–H band at 3310–3400 cm^–1^ disappeared, indicating the insertion of Pb^2+^ into the molecular probe.^84^ Additionally, a new band appeared at approximately 798 cm^–1^, characteristic of metal–nitrogen coordination.^83,84^ These spectral changes confirm the successful stepwise surface functionalization and the subsequent complexation of Pb^2+^ with the molecular probe.

### 4.1 Electrical Signal under Pulsed Temperature Profile

Forming complexes between molecular probes and heavy metal ions requires overcoming a specific activation energy threshold,^85,86^ Figure 3 examines how different pulsed temperature profile conditions affect sensor behavior. In Figure 3A, the sample and substrate were both kept at room temperature, with Pb^2+^ concentrations ranging from 10^–11^ M to 10^–6^ M in 0.02X PBS. Under this condition, detectable signals appeared only at higher concentrations and were generally weak, making low-concentration detection difficult.

**Figure 3:**
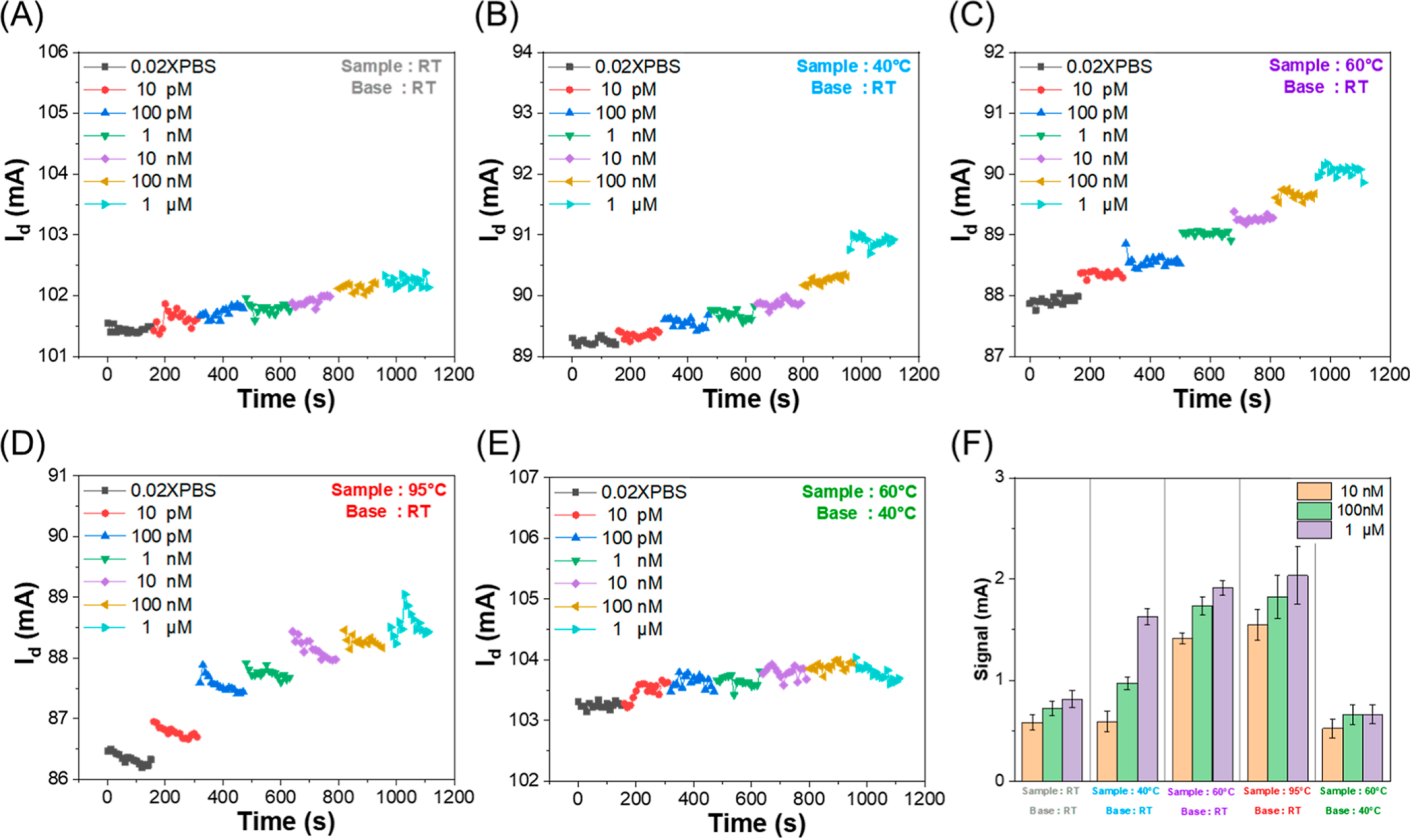
Real-time detection under various temperature conditions. (A) The sample and substrate were kept at ambient conditions. (B–D) Sample temperature increased to 40, 60, and 95 °C, respectively, with the substrate remained at room temperature. (E) Substrate maintained at 40 °C with the sample kept at 60 °C. (F) A bar chart illustrating Pb^2+^ concentration-dependent responses ranging from 10^–8^ M to 10^–6^ M under all tested thermal conditions.

In contrast, Figure 3B–D illustrate the effect of raising the sample temperature to 40 °C, 60 °C, and 95 °C, with the substrate kept at ambient conditions. The sensor showed markedly improved signal responses under these pulsed temperature profile conditions, particularly at 60 °C. However, at 95 °C, the current became unstable, displaying drift and fluctuations due to excessive heating. Figure 3E depicts the outcome when the sample remained at 60 °C and the substrate was heated to 40 °C. In this condition, no signal enhancement was observed; instead, the current exhibited significant fluctuations and drift. This fluctuation is likely due to thermal noise arising from the increased solution temperature,^87,88^ which results from increased random ionic motion that scales with temperature, thereby compromising sensor stability. Figure 3F summarizes the concentration-dependent responses across all thermal conditions, confirming that detection at the picomolar level is achievable and emphasizing that precise temperature control is essential for maximizing sensor sensitivity and performance.

In addition, Figuer S1 presents the results obtained under continuous isothermal heating at 40 °C. Compared to the measurements performed at room temperature (Figure 3A), the signal intensity under this condition is slightly increased; however, no distinct stepwise changes corresponding to concentration variations are observed.

### 4.2 Results of Molecular Dynamics Simulations

This study employed density functional theory (DFT)-based computational methods to systematically investigate the reaction activity and thermodynamic stability of the Pb^2+^–molecular probe complex. As described in eq 8,^89^ the Gibbs free energy (*G*) was calculated by combining the electronic energy (*E*), zero-point energy (ZPE), thermal enthalpy correction (*H*_th_), and entropic contribution (TS), according to the following expression

8 $$G=E+ZPE+H_{th}-TS$$

As shown in Figure 4A, the calculated standard Gibbs free energy change (Δ*G*°) at room temperature is −43.84 kJ·mol^–1^, indicating that the complexation between Pb^2+^ and the molecular probe is thermodynamically spontaneous under standard conditions (Δ*G*° < 0). Despite this favorable thermodynamics, the reaction pathway analysis reveals that the formation of the Pb^2+^–molecular probe complex must overcome an activation energy barrier of 76.32 kJ·mol^–1^, underscoring the critical role of kinetic constraints in governing the overall reaction rate and, consequently, the sensing performance.

**Figure 4:**
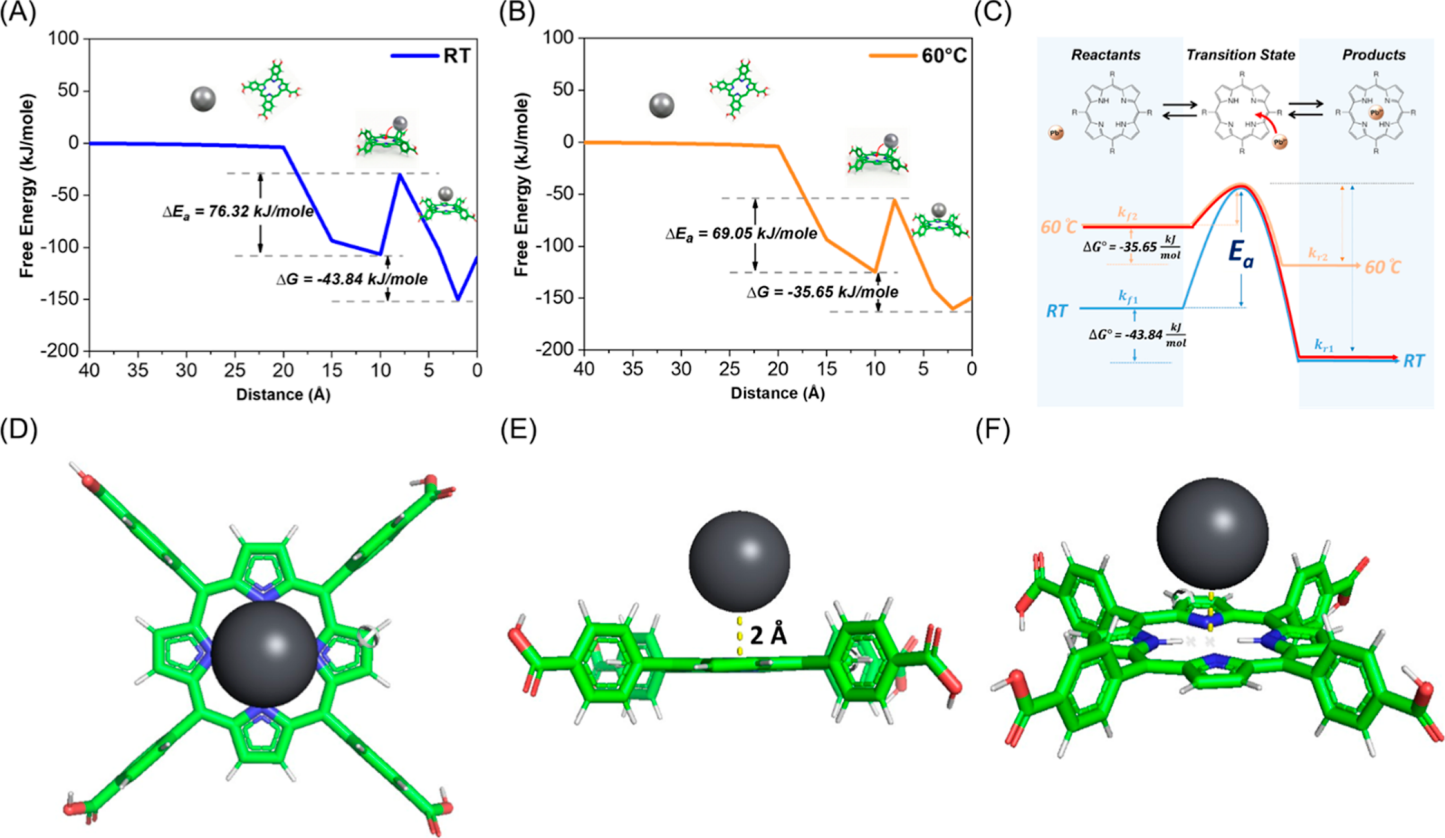
Thermodynamic and kinetic behavior of the molecular probe–Pb­(II) system. (A) Free energy profile at room temperature. (B) Free energy profile at 60 °C. (C) Comparison of free energy profiles at room temperature (blue) and 60 °C (orange) with the pulsed temperature profile used in this study (red). (D) Top view of the formed Pb^2+^–molecular probe complex. (E) Side view of the formed Pb^2+^–molecular probe complex. (F) Bird’s-eye view of the formed Pb^2+^–molecular probe complex.

The reaction behavior at an elevated temperature of 60 °C was further examined, as illustrated in Figure 4B. At this temperature, the calculated standard Gibbs free energy change (Δ*G*°) is −35.65 kJ·mol^–1^, indicating that the reaction remains thermodynamically favorable, although the magnitude is reduced compared to that at room temperature. Concurrently, the activation energy barrier decreases to 69.05 kJ·mol^–1^, suggesting that thermal activation effectively mitigates the kinetic limitations of the complexation process.

Figure 4C systematically presents the reaction kinetics of the molecular probe with Pb^2+^ ions under different temperature conditions. Based on the kinetic parameters extracted from the simulations, the forward reaction rate constants at room temperature (blue) and 60 °C (orange) are denoted as *k*_f1_ and *k*_f2_, respectively. According to the Arrhenius equation^90^ (eq 9), where k is the rate constant, A is the pre-exponential factor, Δ*E*_*a*_ is the activation energy, *T* is the absolute temperature, and *R* is the ideal gas constant, the reaction rate constant increases with increasing temperature or decreasing activation energy. In addition, the reaction rate constants estimated using the Smoluchowski equation^91,92^ (eq 10) (Supporting Information S2), where *G*(*r*) is the Gibbs free energy as a function of the separation distance *r*, *D* is the sum of the diffusion coefficients, *N*_A_ is Avogadro’s constant, *R* is the gas constant, *T* is the absolute temperature, and *a* is the minimum binding distance, exhibit a theoretical trend consistent with the simulation results, namely *k*_f1_ < *k*_f2_. Specifically, the calculated rate constants are *k*_f1_ = 5.33 × 10^4^ s^–1^·M^–1^ and *k*_f2_ = 1.22 × 10^5^ s^–1^·M^–1^. These results indicate that overcoming the activation energy barrier significantly accelerates the complexation reaction between the molecular probe and Pb^2+^ ions.

9 $$k=A\cdot \exp\left(-\Delta E_{a}/RT\right)$$

10 $$k=4\pi DN_{A}/\int_{r_{a}}^{r_{\infty }}\exp\left(G\left(r\right)/RT\right)/r^{2}dr$$

Based on the calculation using eq 11.^93^ and the definition of the dissociation constant (*k*_d_) as the ratio of the forward and reverse reaction rate constants, the reverse reaction rate constant was estimated under continuous isothermal heating conditions without thermal quenching. The results indicate that the reverse reaction rate constant increases markedly with temperature, rising from *k*_*r*1_ = 1.1 × 10^–3^ s^–1^ at room temperature to *k*_*r*2_ = 3 × 10^–1^ s^–1^ at 60 °C. This pronounced increase demonstrates that elevated temperatures significantly accelerate the reverse reaction. Such acceleration compromises the stability of the coordination complex, thereby leading to a reduction in the sensing signal intensity. In contrast, the pulsed temperature profile (red curve) employed in this study effectively enhances the forward reaction rate while maintaining a relatively low reverse reaction rate. As a result, the ratio of the forward to reverse reaction rate constants satisfies *k*_f2_/*k*_*r*1_ > *k*_f1_/*k*_*r*1_ > *k*_f2_/*k*_*r*2_, and this ratio exhibits a positive correlation with the experimentally observed signal intensity.

11 $$k_{d}=\exp\left(-\Delta G^{{}^{\circ}}/RT\right)=k_{r}/k_{f}$$

In addition, Figure 4D–F illustrates the structure of the molecular probe–Pb^2+^ complex obtained. The complex exhibits a nonplanar geometry, with the Pb^2+^ ion positioned approximately 2 Å above the center of the molecular probe.

Based on these results, 10% of the sample treated at 60 °C was introduced into the room-temperature solution, temporarily increasing the substrate temperature (Figure 1F) and thereby accelerating the forward complexation reaction. As the solution subsequently cools back to room temperature, the reverse reaction rate remains relatively low, creating kinetically favorable conditions for complex stabilization. By adopting this pulsed temperature profile, the ratio between the forward and reverse reaction rates is significantly enhanced, leading to improved binding between Pb^2+^ ions and the molecular probe and a stronger sensor response.

In summary, although elevated temperatures can accelerate reaction kinetics, prolonged exposure to high temperatures promotes the reverse reaction, rendering the complexes unstable. To overcome this limitation, the pulsed temperature strategy adopted in this study acts as a “kinetic accelerator”: an initial high temperature rapidly overcomes the activation energy barrier and drives complex formation, followed by rapid cooling to stabilize the products. This approach not only ensures reliable signal responses but also significantly enhances detection sensitivity and stability.

### 4.3 Salt Concentration Effect

This study also examined the influence of salt concentration on the sensor response. Figure 5A–E present real-time detection results under various buffer conditions (0.1X PBS, 0.02X PBS, 0.01X PBS, 0.001X PBS, and 0.0001X PBS), as shown in Figure 5F, signal intensities corresponding to Pb^2+^ concentrations ranging from 10 nM to 1 μM were evaluated under the same temperature profile. The results indicate that at high salt concentration (0.1X PBS), the signal is significantly reduced compared to that at moderate salt concentration (0.01X PBS), due to the ion screening effect.^94^ However, at extremely low salt concentration (0.0001X PBS), a similar attenuation of the signal was observed, accompanied by greater fluctuations in signal stability.

**Figure 5:**
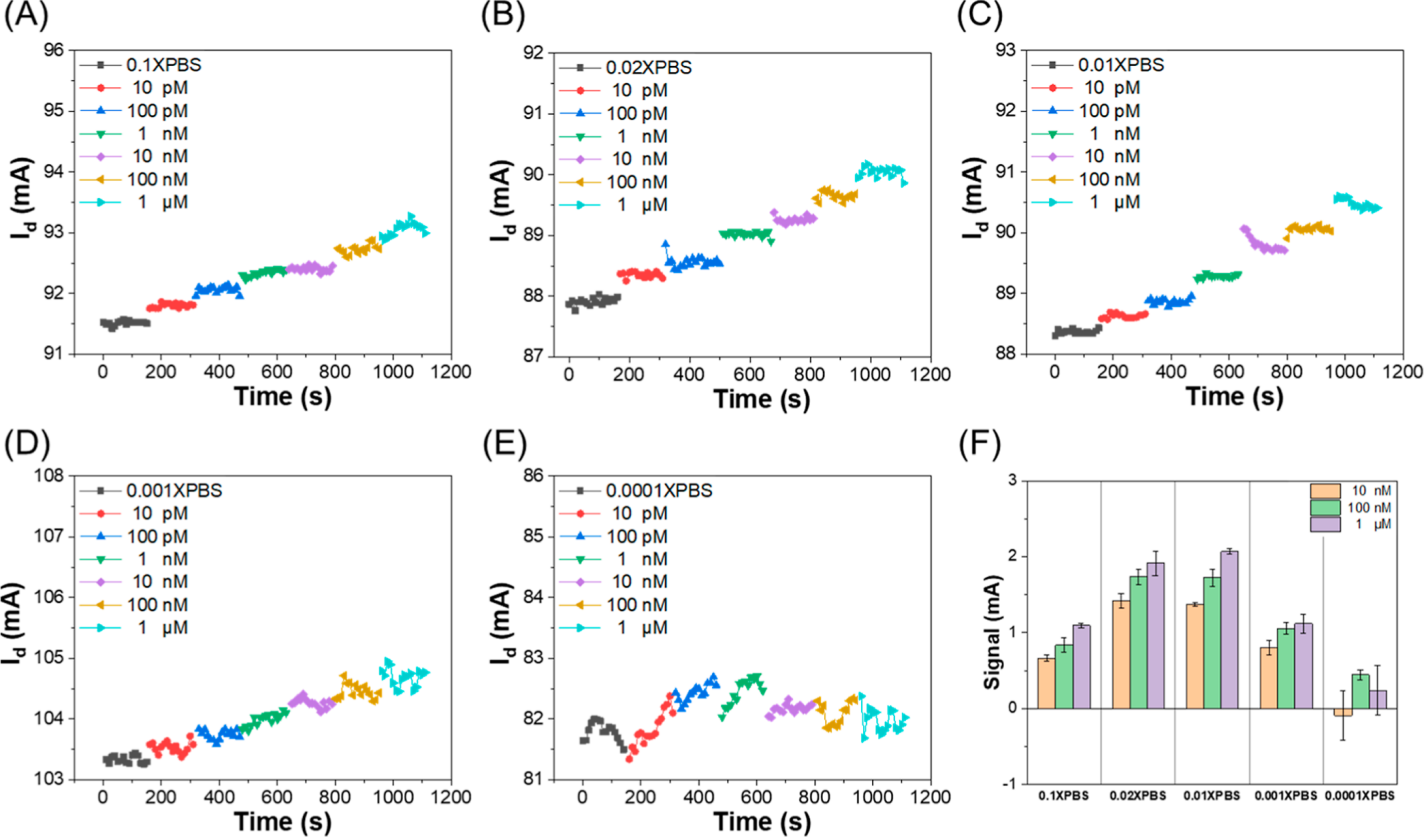
Real-time signal responses at different salt concentrations: (A) 0.1X PBS, (B) 0.02X PBS, (C) 0.01X PBS, (D) 0.001X PBS, and (E) 0.0001X PBS. (F) Signal responses recorded for Pb^2+^ concentrations ranging from 10^–8^ M to 10^–6^ M under these conditions.

This phenomenon can be attributed to the hydration effects of metal ions. In aqueous environments, hydration plays a critical role in the coordination process between anionic ligands and metal ions. When the ionic strength of the solution decreases, metal ions tend to form more stable and thicker hydration shells,^95^ which significantly affect the arrangement and dynamics of the surrounding water molecules.^96,97^ Previous ab initio molecular dynamics (AIMD) and quantum mechanics/molecular mechanics (QM/MM) simulation studies have shown that different metals exhibit distinct hydration numbers and water residence times when coordinated at the molecular probe center,^98,99^ thereby altering their coordination geometry and reaction kinetics. Consequently, the formation of an excessively stable hydration shell around Pb^2+^ may hinder its direct coordination with molecular probes, leading to reduced binding affinity and signal attenuation. A similar trend has been observed in studies examining the competition between metal ion hydration and binding with organic ligands, where a decrease in ionic strength enhances hydration while simultaneously weakening the ability of metal ions to form stable complexes with ligands.^100^

In summary, these results demonstrate that extreme salt concentrations can adversely affect sensor performance. While high ionic strength reduces signals through ion screening, very low ionic strength promotes the formation of stable hydration shells around metal ions, hindering their direct coordination with ligands or sensor surfaces. This dual effect underscores the critical role of metal ion hydration and solution ionic strength in modulating binding affinity and sensor response.

### 4.4 Selectivity Tests

The selectivity of the sensor is shown in Figure 6A, where the current responses of various metal ions at 10^–6^ M are compared, including Ca^2+^, Ni^2+^, Mg^2+^, K^+^, Zn^2+^, Fe^2+^, As^3+^, Cr^3+^, Hg^2+^, Tl^+^, Sn^2+^ and Pb^2+^. Notably, a significant signal change was observed only upon the addition of Pb^2+^, while the other tested metal ions elicited minimal or negligible responses. A comparative analysis of the electrical responses from 14 different heavy metal ions is shown in Figure 6B, showing that the sensor preferentially responds to Pb^2+^. These results indicate that the sensor can effectively distinguish Pb^2+^ from a variety of potentially interfering metal ions, highlighting its potential applicability for selective lead detection in complex sample matrices.

**Figure 6:**
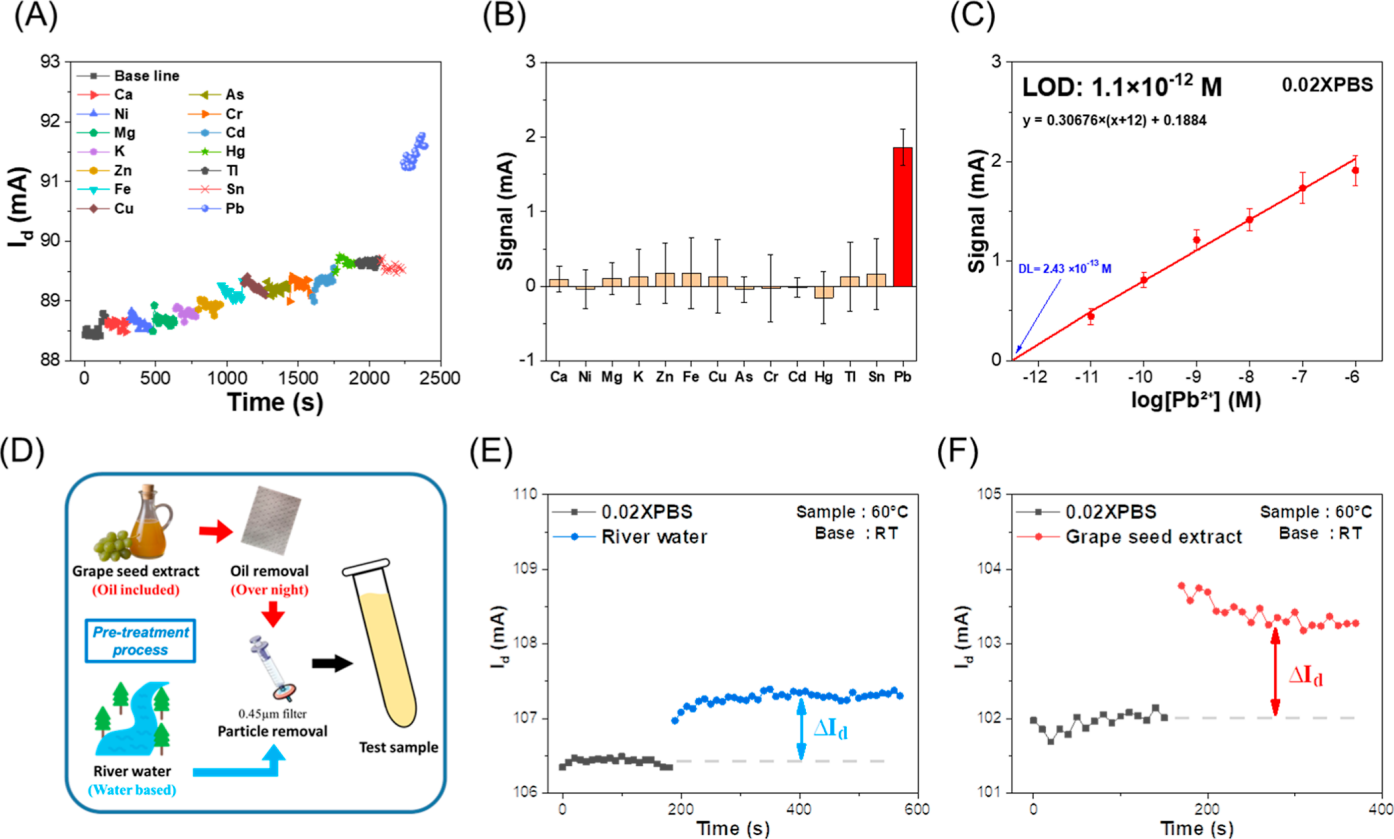
Selectivity tests and real sample detection results of the Bio-EGFET sensing platform. (A) Real-time current responses used to assess selectivity. (B) Bar graph showing the signal responses of 14 different metal ions (*N* = 8) at a concentration of 10^–6^ M. (C) Calibration curve in 0.02X PBS. (D) Pretreatment steps for real samples. (E) Real-time detection signal in river water. (F) Real-time detection signal in grape seed extract.

### 4.5 Real Sample Tests

Figure 6C presents the calibration plot obtained in 0.02X PBS. The LOD of the sensor was calculated using eq 12, where σ = 0.067 mA represents the standard deviation of the blank solution and *S* = 0.307 is the slope of the calibration curve. The LOD corresponds to the intersection point shown in Figure 6C at 0.656 orders of magnitude. Accordingly, the calculated LOD of the sensor is 1.101 × 10^–12^ M.

12 LOD=3σ/S

LOD=3×0.067/0.307=0.656decade

$$LOD=10^{0.656}\times 2.43\times 10^{-13}$$

$$LOD=1.101\times 10^{-12}\;M$$

To validate the analytical performance of the proposed method, a certified reference material (CRM), SLRS-6, provided by the National Research Council Canada, was used. After pH and conductivity calibration, the sample was analyzed, and the results are shown in Figure S2. Based on the calibration curve in Figure 6C, the calculated Pb^2+^ concentration in Supporting Information S3 was 0.182 ppb. The reported values were obtained by averaging the signals from eight sensors (*N* = 8), which agrees well with the certified value of 0.170 ± 0.026 ppb. The deviation was approximately 7%, falling within the variation range of the certified value. These results demonstrate that the proposed sensor enables reliable and accurate quantification of lead in complex matrices.

In addition, the pretreatment workflow for real-world samples, including river water and grape seed oil, is illustrated in Figure 6D. As shown in Figure 6E, the river water sample exhibited a distinct current response. Based on the calibration curve in Figure 6C, the Pb^2+^ concentration was determined to be 0.829 ppb. This value is well below the World Health Organization (WHO) guideline limit for Pb^2+^ in surface water (10 ppb),^101^ indicating that the analyzed river water meets safety standards.

Figure 6F presents the analysis of Pb^2+^ in grape seed extract samples. After oil removal and conductivity adjustment, the sensor’s real-time current response was recorded. The Pb^2+^ concentration was estimated to be 2.024 ppb based on the calibration curve in Figure 6C, which is well below the maximum allowable limit for lead in edible oils established by the Codex Alimentarius Commission (1 mg/kg).^102^ Overall, these results demonstrate that the molecular probe-functionalized sensor enables accurate quantification of Pb^2+^, providing rapid, sensitive, and reliable detection within minutes. This underscores its strong potential for real-time monitoring in environmental and food safety applications.

## 5 Conclusion

This work presents a novel molecular probe-integrated sensing platform capable of rapidly detecting Pb^2+^ ions with exceptional selectivity. The device demonstrated ultrahigh sensitivity, achieving a detection limit of 1 pM, and provided stable, real-time signal responses within seconds. A short DNA linker, endowed with fluorescent functionalities, served as the interfacial bridge, offering flexibility for integration with diverse substrates and surface chemistries. This modular design not only enhances adaptability but also enables direct fluorescence-based monitoring of the surface state. Thermodynamic and kinetic analyses revealed that increasing the sample temperature effectively lowers the reaction’s activation barrier, accelerating Pb^2+^ complexation and improving detection sensitivity. Additionally, variations in ionic strength were found to modulate ion hydration and coordination behavior, influencing both signal reproducibility and binding affinity. Therefore, fine-tuning these experimental parameters is essential for consistent high performance across different environmental conditions. In summary, this study underscores the importance of controlling reaction kinetics, temperature, and ionic environment to optimize molecular probe-based Pb^2+^ sensors. The insights gained provide valuable guidance for designing next-generation heavy metal detection systems with enhanced precision, stability, and adaptability.

## Supplementary Material



## Notes

S1: Continuous constant temperature heating experiment. Figure S1. Real-time current response under continuous constant-temperature heating at 40 °C. S2: Smoluchowski Equation with DFT-Derived Potential of Mean Force. Table S1. DFT-calculated free energies at different Pb^2+^-molecular probe separation distances. Table S2. Calculated diffusion coefficients. Table S3. Summary of calculated parameters and rate constants. S3: Validation Using Certified Reference Materials (CRMs). Figure S2. Current response of the SLRS-6 certified reference material after pH and conductivity calibration (PDF)

## References

[1] Jomova K., Alomar S. Y., Nepovimova E., Kuca K., Valko M. (2025). Heavy metals:
toxicity and human health effects. Arch. Toxicol..

[2] Ijaz, S. ; Iqbal, J. ; Abbasi, B. A. ; Tufail, A. ; Ullah, Z. ; Yaseen, T. ; Ali, I. ; Uddin, S. ; Iqbal, R. Heavy metal–polluted arable land and its consequences: A global scenario. In Biochar-Assisted Remediation of Contaminated Soils Under Changing Climate; Elsevier, 2024; pp 71–99.

[3] Boskabady M., Marefati N., Farkhondeh T., Shakeri F., Farshbaf A., Boskabady M. H. (2018). The effect
of environmental lead exposure on human
health and the contribution of inflammatory mechanisms, a review. Environ. Int..

[4] Collin M. S., Venkatraman S. K., Vijayakumar N., Kanimozhi V., Arbaaz S. M., Stacey R. G. S., Anusha J., Choudhary R., Lvov V., Tovar G. I. (2022). Bioaccumulation of lead
(Pb) and its effects on human: A review. J.
Hazard. Mater. Adv..

[5] Kumar A., Kumar A., Cabral-Pinto M. M. S., Chaturvedi A. K., Shabnam A. A., Subrahmanyam G., Mondal R., Gupta D. K., Malyan S. K., Kumar S. S., Khan A. S. (2020). Lead toxicity: health
hazards, influence on food chain, and sustainable remediation approaches. Int. J. Environ. Res. Public Health.

[6] Hou D., Jia X., Wang L., McGrath S. P., Zhu Y.-G., Hu Q., Zhao F.-J., Bank M. S., O’Connor D., Nriagu J. (2025). Global soil pollution by toxic metals threatens agriculture
and human health. Science.

[7] Li F., Meng L., Ding W., Wang J., Ge L. (2022). Review of
energy-dispersive X-ray fluorescence on food elements detection. X-Ray Spectrom..

[8] Alam M. A., Tiwari M., Trivedi A., Khooha A., Singh A. (2021). Labeling elemental
detection sensitivities in part per billion range using conventional
geometry synchrotron assisted EDXRF measurements. arXiv.

[9] Sumarno D., Kusumaningtyas D. I. (2019). Penentuan Limit Deteksi dan Limit
Kuantitasi untuk
Analisis Logam Timbal (Pb) dalam Air Tawar Atom Menggunakan Spektrofotometer
Serapan. Buletin Teknik Litkayasa Sumber Daya
dan Penangkapan.

[10] Zhuravlev A., Zacharia A., Arabadzhi M., Turetta C., Cozzi G., Barbante C. (2016). Comparison of analytical methods: ICP-QMS, ICP-SFMS
and FF-ET-AAS for the determination of V, Mn, Ni, Cu, As, Sr, Mo,
Cd and Pb in ground natural waters. Int. J.
Environ. Anal. Chem..

[11] Abdelmonem B. H., Kamal L. T., Elbaz R. M., Khalifa M. R., Abdelnaser A. (2025). From contamination
to detection: The growing threat of heavy metals. Heliyon.

[12] Zhu N., Liu X., Peng K., Cao H., Yuan M., Ye T., Wu X., Yin F., Yu J., Hao L. (2022). A novel
aptamer-imprinted polymer-based electrochemical biosensor for the
detection of lead in aquatic products. Molecules.

[13] Qian S., Han Y., Xu F., Feng D., Yang X., Wu X., Hao L., Yuan M. (2022). A fast, sensitive, low-cost electrochemical paper-based
chip for real-time simultaneous detection of cadmium (II) and lead
(II) via aptamer. Talanta.

[14] Zhang Z., Karimi-Maleh H., Wen Y., Darabi R., Wu T., Alostani P., Ghalkhani M. (2023). Nanohybrid of antimonene@ Ti3C2Tx-based
electrochemical aptasensor for lead detection. Environ. Res..

[15] Zhang Z., Karimi-Maleh H. (2023). Label-free
electrochemical aptasensor based on gold
nanoparticles/titanium carbide MXene for lead detection with its reduction
peak as index signal. Adv. Compos. Hybrid Mater..

[16] Liu C., Wang Y., Li Y., Meng S., Li W., Liu D., You T. (2023). Electric field-enabled
aptasensing interfacial engineering
to simultaneously enhance specific preconcentration and electrochemical
detection of mercury and lead ions. Sci. Total
Environ..

[17] Huang Z., Song H., Feng L., Qin J., Wang Q., Guo B., Wei L., Lu Y., Guo H., Zhu D. (2023). A novel ultrasensitive electrochemical sensor
based on a hybrid of
rGO/MWCNT/AuNP for the determination of lead (II) in tea drinks. Microchem. J..

[18] Zhang L., Wu J., Xiao M., Zhang S., Ren S., Luo D., Xi F., Liu H., Li Y., Li Q. (2024). Aptamer-functionalized
gold nanoparticles for fast and selective electrochemical sensing
of lead in tobacco. Int. J. Electrochem. Sci..

[19] Mondragon-Sanchez J., Liquier J., Shafer R., Taillandier E. (2004). Tetraplex
structure formation in the thrombin-binding DNA aptamer by metal cations
measured by vibrational spectroscopy. J. Biomol.
Struct. Dyn..

[20] Khoshbin Z., Housaindokht M. R., Izadyar M., Bozorgmehr M. R., Verdian A. (2020). The investigation of
the G-quadruplex aptamer selectivity
to Pb2+ ion: a joint molecular dynamics simulation and density functional
theory study. J. Biomol. Struct. Dyn..

[21] Li M., Shen G., Ding Y., Gong Z., Zhou Y., Chen Y. (2025). Fullerene-based PEC
aptasensor with DNA super sandwich structures
and Pb2+-G quadruplex structures. Microchem.
J..

[22] Bhattacharyya D., Mirihana Arachchilage G., Basu S. (2016). Metal cations in G-quadruplex
folding and stability. Front. Chem..

[23] Zeng G.-C., Huang H.-W., Lin C.-K., Chen J.-C., Dong G.-C., Hung S.-C., Wang Y.-L. (2025). Design and demonstration
of a temperature-resistant
aptamer structure for highly sensitive mercury ion detection with
BioFETs. Talanta.

[24] Tan S. Y., Acquah C., Tan S. Y., Ongkudon C. M., Danquah M. K. (2017). Characterisation
of charge distribution and stability of aptamer-thrombin binding interaction. Process Biochem..

[25] Hianik T., Ostatná V., Sonlajtnerova M., Grman I. (2007). Influence of ionic
strength, pH and aptamer configuration for binding affinity to thrombin. Bioelectrochemistry.

[26] Mao C., Robinson K. J., Yuan D., Bakker E. (2022). Ion–ionophore
interactions in polymeric membranes studied by thin layer voltammetry. Sens. Actuators, B.

[27] Brasseur R., Notredame M., Ruysschaert J.-M. (1983). Lipid-water interface mediates reversible
ionophore conformational change. Biochem. Biophys.
Res. Commun..

[28] Mamardashvili G., Mamardashvili N., Koifman O. (2021). Macrocyclic receptors for identification
and selective binding of substrates of different nature. Molecules.

[29] Kim S., Lee D. H., Park K.-M., Jung J. H., Lee S. S., Park I.-H. (2022). Unexpected Solvent-Dependent
Self-Assembly of Alkali
Metal Complexes of Calix [6]-mono-crown-4: Dinuclear Bowls, a Pseudo-Capsule,
and a One-Dimensional Polymer. Inorg. Chem..

[30] Tsivadze A. Y., Baulin V., Kostikova G., Bezdomnikov A. (2023). Selective
extraction of lithium from mineral, hydromineral, and secondary raw
materials. Herald Russ. Acad. Sci..

[31] Feng Y.-C., Wang X., Wang D. (2023). Metal porphyrins
and metal phthalocyanines
as designable molecular model electrocatalysts. Mater. Chem. Front..

[32] Wang D., Wang J., Gao X. J., Ding H., Yang M., He Z., Xie J., Zhang Z., Huang H., Nie G. (2024). Employing noble metal–porphyrins to engineer robust and highly
active single-atom nanozymes for targeted catalytic therapy in nasopharyngeal
carcinoma. Adv. Mater..

[33] Chen J., Zhu Y., Kaskel S. (2021). Porphyrin-based metal–organic frameworks for
biomedical applications. Angew. Chem., Int.
Ed..

[34] Isaeva V., Gamov G., Sharnin V. (2021). Quantum-Chemical
Calculations and
Stability Analysis of Copper (II) Complexes with Cryptand [2.2. 2]. Russ. J. Inorg. Chem..

[35] Sachdeva G., Bamal Y., Ladan A., Tiwari O. S., Rawat V., Yadav P., Verma V. P. (2023). Calixarene-metal
complexes in lactide
polymerization: The story so far. ACS Omega.

[36] Mei C. J., Ahmad S. A. A. (2021). A review on the
determination heavy metals ions using
calixarene-based electrochemical sensors. Arabian
J. Chem..

[37] Ohto K. (2021). Review of
adsorbents incorporating calixarene derivatives used for metals recovery
and hazardous ions removal: the concept of adsorbent design and classification
of adsorbents. J. Inclusion Phenom. Macrocyclic
Chem..

[38] Abd
Karim N. F. N., Supian F. L., Musa M., Ayop S. K., Azmi M. S., Yazid M. D., Yi W. Y. (2023). Calixarene derivatives:
a mini-review on their synthesis and demands in nanosensors and biomedical
fields. Mini Rev. Med. Chem..

[39] Vögtle F. (1980). New ligand
systems for ions and molecules-and electronic effects upon complexation. Pure Appl. Chem..

[40] Kariuki B. M., Lee S.-O., Harris K. D., Kim H.-S., Do K.-S., Kim K.-I. (2002). Structural rationalization of a highly selective ammonium
ionophore. Cryst. Growth Des..

[41] Farahani M. D., Montealegre I. M., Gilavan M. T., Kirby T., Selvaganapathy P. R., Kruse P. (2024). A highly sensitive ion-selective chemiresistive sensor for online
monitoring of lead ions in water. Analyst.

[42] Fan Y., Xu Z., Huang Y., Wang T., Zheng S., DePasquale A., Brüeckner C., Lei Y., Li B. (2020). Long-term continuous
and real-time in situ monitoring of Pb (II) toxic contaminants in
wastewater using solid-state ion selective membrane (S-ISM) Pb and
pH auto-correction assembly. J. Hazard. Mater..

[43] Silva R., Zhao K., Ding R., Chan W. P., Yang M., Yip J. S. Q., Lisak G. (2022). Ion-selective membrane
modified microfluidic
paper-based solution sampling substrates for potentiometric heavy
metal detection. Analyst.

[44] Liu Y., Wang X., Zeng X., Waterhouse G. I. N., Jiang X., Zhang Z., Yu L. (2023). Antifouling improvement
in Pb2+ ion selective electrodes by using an environmentally friendly
capsaicin derivative. Talanta.

[45] Liu Y., Waterhouse G. I., Jiang X., Zhang Z., Yu L. (2024). A cathodically
polarized PANI-based lead ion-selective electrode: achieving high
stability with antibiofouling capabilities. Microchim. Acta.

[46] Jiang W., Liu C., Zhao Y., Waterhouse G. I. N., Zhang Z., Yu L. (2019). A solid-contact
Pb2+-selective electrode based on a hydrophobic polyaniline microfiber
film as the ion-to-electron transducer. Synth.
Met..

[47] Liu Y., Zeng X., Waterhouse G. I. N., Jiang X., Zhang Z., Yu L. (2023). Potential stability
improvement in solid-contact Pb2+ ion-selective
electrodes by using polyaniline/montmorillonite composites as the
ion-to-electron transducer. J. Electroanal.
Chem..

[48] Wardak C., Morawska K., Paczosa-Bator B., Grabarczyk M. (2023). Improved lead
sensing using a solid-contact ion-selective electrode with polymeric
membrane modified with carbon nanofibers and ionic liquid nanocomposite. Materials.

[49] Chen Y.-T., Hseih C.-Y., Sarangadharan I., Sukesan R., Lee G.-Y., Chyi J.-I., Wang Y.-L. (2018). Beyond
the limit of ideal nernst
sensitivity: ultra-high sensitivity of heavy metal ion detection with
ion-selective high electron mobility transistors. ECS J. Solid State Sci. Technol..

[50] Wang S.-L., Hsieh C.-Y., Wang Y.-L. (2019). FET Based Heavy Metal Ion Selective
Sensors for Lead Ion Detection in Whole Blood. ECS Trans..

[51] Chen Y.-T., Sarangadharan I., Sukesan R., Hseih C.-Y., Lee G.-Y., Chyi J.-I., Wang Y.-L. (2018). High-field modulated ion-selective
field-effect-transistor (FET) sensors with sensitivity higher than
the ideal Nernst sensitivity. Sci. Rep..

[52] Wang S.-L., Hsieh C.-Y., Sukesan R., Chen J.-C., Wang Y.-L. (2020). Highly
sensitive lead ion detection in one drop of human whole blood using
impedance-modulated field-effect transistors and a portable measurement
device. ECS J. Solid State Sci. Technol..

[53] Lee H. K., Song K., Seo H. R., Choi Y.-K., Jeon S. (2004). Lead­(II)-selective
electrodes based on tetrakis­(2-hydroxy-1-naphthyl)­porphyrins: the
effect of atropisomers. Sens. Actuators, B.

[54] Miyahara Y., Yamashita K., Ozawa S., Watanabe Y. (1996). Shift and drift of
electromotive forces of solid-state electrodes with ion-selective
liquid membranes. Anal. Chim. Acta.

[55] Fibbioli M., Morf W. E., Badertscher M., de Rooij N. F., Pretsch E. (2000). Potential
drifts of solid-contacted ion-selective electrodes due to zero-current
ion fluxes through the sensor membrane. Electroanalysis.

[56] Malon A., Bakker E., Pretsch E. (2007). Backside calibration
potentiometry:
Ion activity measurements with selective supported liquid membranes
by calibrating from the inner side of the membrane. Anal. Chem..

[57] Mele L. J., Palestri P., Selmi L., Alam M. A. (2022). Modeling non-equilibrium
ion-transport in ion-selective-membrane/electrolyte interfaces for
electrochemical potentiometric sensors. IEEE
Sens. J..

[58] Alharthi S. S., Fallatah A. M., Al-Saidi H. M. (2021). Design and characterization of electrochemical
sensor for the determination of mercury (II) ion in real samples based
upon a new schiff base derivative as an ionophore. Sensors.

[59] Wang, S.-L. ; Sukesan, R. ; Sarangadharan, I. ; Wang, Y.-L. FET based heavy metal ion sensor to detect mercury ion from waste water. In2019 20th International Conference on Solid-State Sensors, Actuators and Microsystems & Eurosensors XXXIII (TRANSDUCERS & EUROSENSORS XXXIII); IEEE, 2019; pp 1270–1273 .

[60] Goldberg J. M., Speight L. C., Fegley M. W., Petersson E. J. (2012). Minimalist
probes for studying protein dynamics: thioamide quenching of selectively
excitable fluorescent amino acids. J. Am. Chem.
Soc..

[61] Mou C., Chen D., Wang X., Zhang B., He T., Xin H., Liu F.-c. (1991). Surface-enhanced
Raman scattering of TSPP, Ag­(II)­TSPP,
and Pb­(II)­TSPP adsorbed on AgI and AGCl colloids. Spectrochim. Acta, Part A.

[62] Ohtomo T., Yokoyama A., Konno M., Ohno O., Igarashi S., Takagai Y. (2016). β-Cyclodextrin as a metal-anionic
porphyrin complexation
accelerator in aqueous media. Anal. Sci..

[63] Kawamura K., Igarashi S., Yotsuyanagi T. (2007). Stopped-flow
spectrophotometric determination
of nM level of Pb (II) using 5, 10, 15, 20-terakis (1-methylpyridinium-4-yl)
porphine. Microchim. Acta.

[64] Zamadar M., Orr C., Uherek M. (2016). Water soluble
cationic porphyrin sensor for detection
of Hg2+, Pb2+, Cd2+, and Cu2+. J. Sens..

[65] Wang Q., Ke W., Lou H., Han Y., Wan J. (2021). A novel fluorescent
metal-organic framework based on porphyrin and AIE for ultra-high
sensitivity and selectivity detection of Pb2+ ions in aqueous solution. Dyes Pigm..

[66] Radi S., Abiad C. E., Moura N. M. M., Faustino M. A. F., Neves M. G. P. M. S. (2019). New hybrid
adsorbent based on porphyrin functionalized silica for
heavy metals removal: Synthesis, characterization, isotherms, kinetics
and thermodynamics studies. J. Hazard. Mater..

[67] Dang L. X. (1995). Mechanism
and thermodynamics of ion selectivity in aqueous solutions of 18-crown-6
ether: a molecular dynamics study. J. Am. Chem.
Soc..

[68] Najjari B., Le Gac S., Roisnel T., Dorcet V., Boitrel B. (2012). Metal Migration
Processes in Homo-and Heterobimetallic Bismuth (III)–Lead (II)
Porphyrin Complexes: Emergence of Allosteric Newton’s Cradle-like
Devices. J. Am. Chem. Soc..

[69] Elimelech, M. ; Gregory, J. ; Jia, X. ; Williams, R. Particle Deposition and Aggregation: Measurement, Modelling and Simulation; Elsevier, 1995; pp 9–32.

[70] Bergveld, P. ISFET, theory and practice. In IEEE Sensor Conference; Institute of Electrical and Electronics Engineers: Toronto, 2003..

[71] Sposito, G. Gouy-chapman theory. In Encyclopedia of Geochemistry; Springer, 2018; pp 623–628.

[72] Du H., Lin X., Xu Z., Chu D. (2015). Electric double-layer transistors:
a review of recent progress. J. Mater. Sci..

[73] Tsai S.-Y., Huang C.-C., Chen P.-H., Tripathi A., Wang Y.-R., Wang Y.-L., Chen J.-C. (2022). Rapid drug-screening
platform using
field-effect transistor-based biosensors: A study of extracellular
drug effects on transmembrane potentials. Anal.
Chem..

[74] Wu C.-R., Wang S.-L., Chen P.-H., Wang Y.-L., Wang Y.-R., Chen J.-C. (2021). Demonstration
of the enhancement of gate bias and ionic
strength in electric-double-layer field-effect-transistor biosensors. Sens. Actuators, B.

[75] Liao L.-W., Chen P.-H., Tsai S.-Y., Tripathi A., Paulose A. K., Chang S.-J., Wang Y.-L. (2021). Rapid β-human
chorionic gonadotropin
detection in urine with electric-double-layer gated field-effect transistor
biosensors and a handheld device. Biomicrofluidics.

[76] Neese F., Wennmohs F., Becker U., Riplinger C. (2020). The ORCA quantum
chemistry program package. J. Chem. Phys..

[77] Whelan D. R., Bambery K. R., Puskar L., McNaughton D., Wood B. R. (2013). Quantification of DNA in simple eukaryotic cells using
Fourier transform infrared spectroscopy. J.
Biophotonics.

[78] Meade A. D., Lyng F. M., Knief P., Byrne H. J. (2007). Growth substrate
induced functional changes elucidated by FTIR and Raman spectroscopy
in in–vitro cultured human keratinocytes. Anal. Bioanal. Chem..

[79] Mehra S., Chadha P. (2020). Alterations in structure of biomolecules
using ATR-FTIR
and histopathological variations in brain tissue of Channa punctatus
exposed to 2Naphthalene sufonate. Toxicol. Res..

[80] de
Souza N. M. P., da Rosa D. K. A., de Moraes C., Caeran M., Hoffmann M. B., Aita E. P., Prochnow L., da Motta A. L. A., Corbellini V. A., Rieger A. (2024). Structural characterization
of DNA amplicons by ATR-FTIR spectroscopy as a guide for screening
metainflammatory disorders in blood plasma. Spectrochim. Acta, Part A.

[81] Sarker M. Z., Rahman M. M., Minami H., Suzuki T., Ahmad H. (2022). Amine functional
silica–supported bimetallic Cu-Ni nanocatalyst and investigation
of some typical reductions of aromatic nitro-substituents. Colloid Polym. Sci..

[82] Yao B., Peng C., He Y., Zhang W., Zhang Q., Zhang T. (2016). Conjugated microspheres FeTCPP–TDI–TiO2 with enhanced
photocatalytic performance for antibiotics degradation under visible
light irradiation. Catal. Lett..

[83] Wu J., Wang W., Wang Z. (2020). Porphin-based
carbon dots for “turn
off–on” phosphate sensing and cell imaging. Nanomaterials.

[84] Fidalgo-Marijuan A., Barandika G., Bazán B., Urtiaga M.-K., Arriortua M.-I. (2011). Self-assembly
of iron TCPP (meso-tetra­(4-carboxyphenyl)­porphyrin) into a chiral
2D coordination polymer. Polyhedron.

[85] Shen Y., Ryde U. (2005). Reaction mechanism
of porphyrin metallation studied by theoretical
methods. Chem. Eur J..

[86] Pukhovskaya S., Efimovich V., Golubchikov O. (2013). Effect of
structural and electronic
properties of substituents on the metal porphyrin formation kinetics. Russ. J. Inorg. Chem..

[87] Kuo W.-C., Sarangadharan I., Pulikkathodi A. K., Chen P.-H., Wang S.-L., Wu C.-R., Wang Y.-L. (2019). Investigation
of electrical stability
and sensitivity of electric double layer gated field-effect transistors
(FETs) for miRNA detection. Sensors.

[88] Zhang D., Must I., Netzer N. L., Xu X., Solomon P., Zhang S.-L., Zhang Z. (2016). Direct assessment of solid–liquid
interface noise in ion sensing using a differential method. Appl. Phys. Lett..

[89] Feng Z., Hu C., Tang H., Shen K., Chen L., Li Y. (2025). Dual-atomic
Cu–Ag pairs boosting selective electroreduction of CO 2 to
acetate. Chem. Sci..

[90] Crapse J., Pappireddi N., Gupta M., Shvartsman S. Y., Wieschaus E., Wühr M. (2021). Evaluating the Arrhenius equation
for developmental processes. Mol. Syst. Biol..

[91] Rice, S. A. Diffusion-Limited Reactions; Elsevier, 1985.

[92] Smoluchowski M. v. (1918). Versuch
einer mathematischen Theorie der Koagulationskinetik kolloider Lösungen. Z. Phys. Chem..

[93] Decherchi S., Cavalli A. (2020). Thermodynamics and kinetics of drug-target binding
by molecular simulation. Chem. Rev..

[94] Thriveni G., Ghosh K. (2022). Advancement and challenges
of biosensing using field effect transistors. Biosensors.

[95] Song Y., Ganguly A., Bradley Z., Lee D., Bhalla N. (2025). Nanoplasmonics
Reveal Ionic-Strength-Driven Hydration of Nanoparticles. Adv. Funct. Mater..

[96] Persson I. (2010). Hydrated metal
ions in aqueous solution: How regular are their structures?. Pure Appl. Chem..

[97] Kim Y., Choi J. (2025). Atomistic understanding of hydration shell mechanics
modulating freezing
dynamics of alkali chloride aqueous solution. Desalination.

[98] Moin S. T., Hofer T. S. (2016). Zinc-and copper-porphyrins
in aqueous solution–two
similar complexes with strongly contrasting hydration. Mol. BioSyst..

[99] Jamal S., Naz Z., Moin S. T., Hofer T. S. (2023). Deciphering Structural and Dynamical
Properties of Hydrated Cobalt Porphyrins via Ab Initio Quantum Mechanical
Charge Field Molecular Dynamics Simulation. J. Phys. Chem. B.

[100] Ohtaki H., Radnai T. (1993). Structure and dynamics of hydrated
ions. Chem. Rev..

[101] Edition, F. Guidelines for drinking-water quality; World Health Organization, 2011, 38; pp 104–108.

[102] González-Torres P., Puentes J. G., Moya A. J., La Rubia M. D. (2023). Comparative Study of the Presence
of Heavy Metals in
Edible Vegetable Oils. Appl. Sci..

