# Supplementary material for: Highly Improved Sensitivity of FET Sensors Based on a Pulsed Temperature Profile for Lead Ion Detection
Source: Anal Chem. 2026 Apr 7;98(20):14748–61. doi: 10.1021/acs.analchem.5c07438 (PMC13217370; doi:10.1021/acs.analchem.5c07438)
Supplement: Supplementary file 1 [file ac5c07438_si_001.pdf]

Supplementary information

Highly Improved Sensitivity of FET Sensors Based on a Pulsed Temperature Profile for Lead Ion Detection

Guan-Cheng Zeng[1], She Yi-Te[1], Ching-Yao Lan[2], Yi-Fang Wu[1], Chia-Kai Lin[1], Hsuan-Wei Huang[3], Jung-Chih Chen[4], Sheng-Chun Hung[5], Guo-Chun Dong[6], and Yu-Lin Wang [1,2,7*]

[1]Institute of Nanoengineering and Microsystems, National Tsing Hua University, Hsinchu, Taiwan.

[2]Department of Power Mechanical Engineering, National Tsing Hua University, Hsinchu, Taiwan

[3]Department of Medical Laboratory Science and Biotechnology, Central Taiwan University of Science and Technology, Taichung, Taiwan

[5]Department of Electrical Engineering, Feng Chia University, Taichung, Taiwan

[4]Institute of Biomedical Engineering, National Yang Ming Chiao Tung University, Hsinchu, Taiwan

[6]Institute of Biomedical Engineering and Nanomedicine, National Health Research Institutes, Miaoli, Taiwan

[7]College of Semiconductor Research, National Tsing Hua University, Hsinchu, Taiwan

**Corresponding Author: Yu-Lin Wang, e-mail:* ylwang@mx.nthu.edu.tw

S1. Continuous constant temperature heating experiment

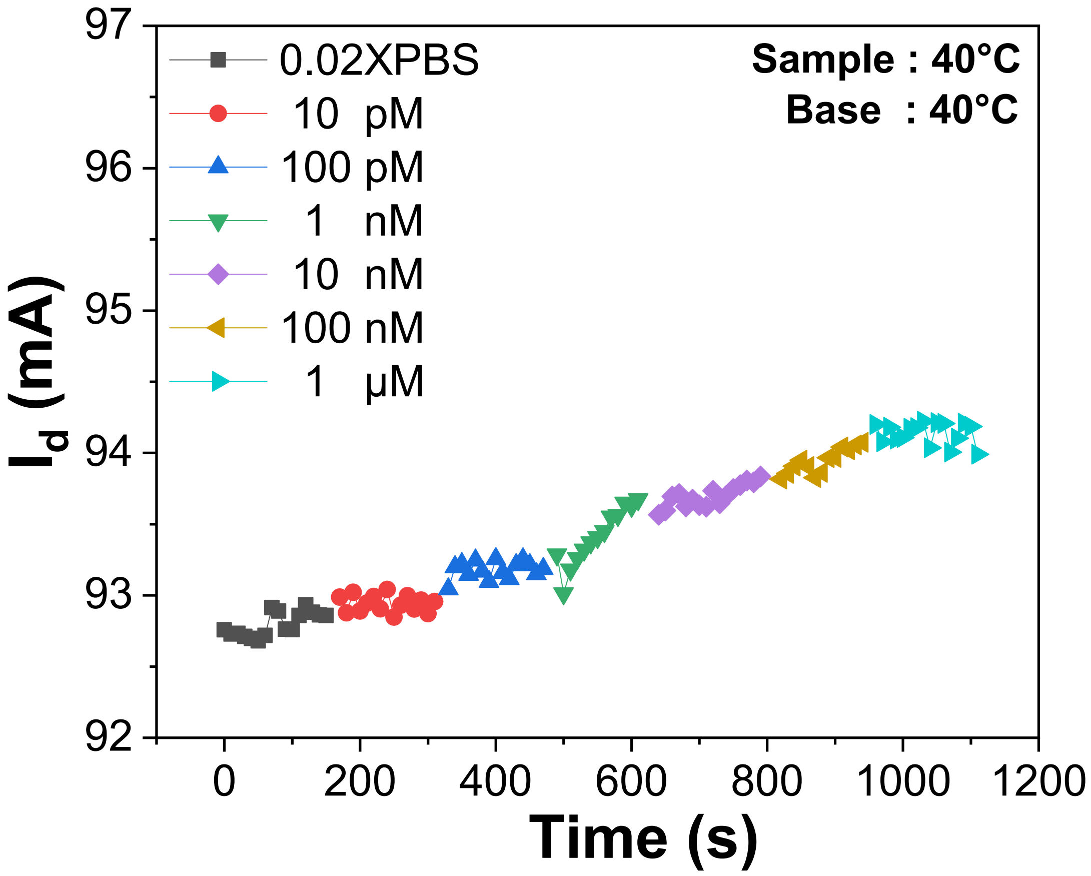

Figure S1. Real-time current response under continuous constant-temperature heating at 40 ˚C.

S2. Smoluchowski Equation with DFT-Derived Potential of Mean Force

➢ Theoretical Background

The association rate constant (k_f) for diffusion-controlled bimolecular reactions can be calculated using the Smoluchowski equation [1, 2] modified to include a potential of mean force (PMF):

$$k_f = 4\pi D N_A / \int_{r_a}^{r_\infty} \exp\left(G(r)/RT\right)/r^2 \, dr \qquad \text{(S1)}$$

where:

- G(r) is the potential of mean force (PMF), expressed as the Gibbs free energy as a function of separation distance r, obtained from DFT calculations with implicit solvation.
- $D = D_A + D_B$ is the sum of diffusion coefficients
- N_A is Avogadro's constant (6.022×10^{23} mol^{-1})
- R is the gas constant (8.314 J mol^{-1} K^{-1})
- T is temperature in Kelvin
- a is the minimum binding distance (~2 Å)

This approach is superior to the standard Smoluchowski equation, which assumes no interactions between particles (U = 0). By incorporating the complete free energy landscape, G(r), derived from quantum chemical calculations, our method captures both long-range electrostatic interactions and short-range binding effects, as well as the influence of the solvent on the effective interaction potential.

➢ DFT Free Energy Calculations

1. Electronic Energies

The electronic energies E(r) were calculated at the B3LYP/def2-SVP level of theory with SMD implicit solvation model for water at 298 K and 333 K. The Pb^{2+}-molecular probe complex was optimized at various separation distances from 0 to 40 Å. All energies were referenced to the separated state at 40 Å (E = 0 kJ/mol).

2. Entropy Corrections

The translational entropy loss upon binding was estimated using the Sackur-Tetrode equation [3]. For a bimolecular association in solution, approximately one-third of the translational entropy is lost upon complex formation. The entropy correction was calculated as:

$$\Delta S_{trans} = -R \ln(V/N_A) - (3/2)R \ln(2\pi mkT/h^2) - (5/2)R \qquad \text{(S2)}$$

At 298 K, this gives $T\Delta S \approx 36.3$ kJ/mol. This correction was applied to bound states (r < 10 Å) but not to separated states (r > 10 Å).

3. Free Energy Profile

The Gibbs free energy was calculated as $G(r) = E(r) - T\Delta S(r)$, where the entropy correction depends on the binding state. Table S1 presents the complete free energy profile used in the calculations.

Table S1. DFT-calculated free energies at different Pb^{2+}-MOLECULAR PROBE separation distances.

Distance (Å)	G at 298K (kJ/mol)	G at 333K (kJ/mol)
0	-106.75	-109.53
2	-147.63	-150.41
4	-98.72	-101.50
8	-28.48	-30.24
10	-105.31	-106.57
15	-93.57	-93.57
20	-3.85	-3.85
25	-2.24	-2.24
30	-1.23	-1.23
35	-0.52	-0.52
40	0.00	0.00

All energies are referenced to the separated state at 40 Å. The deep minimum at r = 2 Å (ca. -148 kJ/mol) corresponds to the optimal coordination geometry of Pb^{2+} within the MOLECULAR PROBE porphyrin ring.

➢ Diffusion Coefficients

1. Stokes-Einstein Equation

Diffusion coefficients were calculated using the Stokes-Einstein equation [4]:

$$D = k_B T / (6\pi\eta r) \qquad \text{(S3)}$$

where k_B is the Boltzmann constant (1.381×10^{-23} J/K), η is the dynamic viscosity of water, and r is the effective hydrodynamic radius of the species.

2. Parameters and Results

The following parameters were used:

- Pb^{2+} ionic radius: 1.5 Å
- MOLECULAR PROBE molecular radius: 8.0 Å (estimated from molecular structure)
- Water viscosity at 298 K: 8.9×10^{-4} Pa·s
- Water viscosity at 333 K: 4.7×10^{-4} Pa·s

Table S2. Calculated diffusion coefficients.

Temperature	D(Pb^{2+}) (m^2/s)	D(MOLECULAR PROBE) (m^2/s)	D(total) (m^2/s)
298 K	1.64×10^{-9}	3.07×10^{-10}	1.94×10^{-9}
333 K	3.47×10^{-9}	6.50×10^{-10}	4.12×10^{-9}

The diffusion coefficient increases approximately 2.1-fold from 298 K to 333 K, primarily due to the decrease in water viscosity at elevated temperature.

➢ Numerical Integration Procedure

1. Cubic Spline Interpolation

The DFT data provides G(r) at 11 discrete distances. To evaluate the integral in Equation S1, cubic spline interpolation was used to create a continuous function. The natural cubic spline ensures continuity of the function and its first two derivatives, providing smooth interpolation between data points.

2. Integration Method

The integral $I = \int_{r_a}^{r_\infty} \exp(G(r)/RT)/r^2 dr$ was evaluated numerically using adaptive Gaussian quadrature with the following specifications:

- Lower limit: a = 2 Å (minimum binding distance)
- Upper limit: 40 Å (where G(r) ≈ 0)
- Method: scipy.integrate.quad with automatic subdivision
- Convergence criterion: relative error $< 10^{-6}$

Analysis of the integrand $\exp(G(r)/RT)/r^2$ reveals that the main contribution to the integral comes from long-range distances (r > 20 Å) where G(r) ≈ 0. The deep binding well at short distances (r < 10 Å) contributes

minimally due to the exponentially small values of exp[G(r)/RT]. This indicates that the rate is diffusion-controlled, with the strong binding energy serving to efficiently capture species once they approach closely.

- **Calculated Association Rate Constants**

Table S3 summarizes the calculated association rate constants and key parameters at both temperatures.

Parameter	298 K	333 K	Ratio (333K/298K)
D(total) (m^2/s)	1.94×10^{-9}	4.12×10^{-9}	2.12
Integral I (m^{-1})	1.38×10^{8}	1.28×10^{8}	0.93
k_f ($M^{-1}s^{-1}$)	5.33×10^{4}	1.22×10^{5}	2.29

Table S3. Summary of calculated parameters and rate constants.

- The 2.29-fold increase in k_f from 298 K to 333 K results from two factors: (1) increased diffusion rate due to lower viscosity (2.12-fold), and (2) slight decrease in the integral due to reduced relative barrier height at higher thermal energy (0.93-fold). The overall temperature dependence is dominated by the diffusion enhancement.

S3. Validation Using Certified Reference Materials (CRMs)

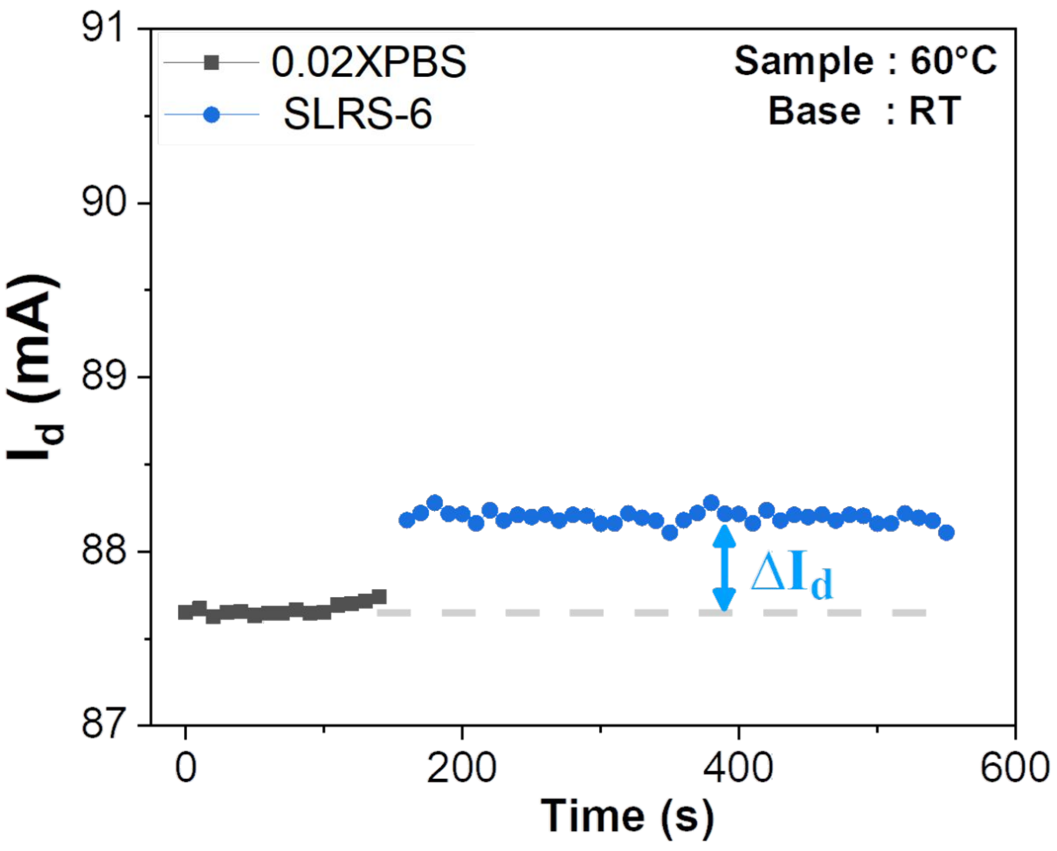

Figure S2. Current response of the SLRS-6 certified reference material after pH and conductivity calibration.

Figure S2 shows the current response of the SLRS-6 certified reference material. The average current change (ΔI_d) obtained from eight sensors (N = 8) was 0.598 mA. Based on the calibration curve equation shown in Figure 6C:

$$y = 0.30676 \times (x + 12) + 0.1884$$

where ΔI_d = y = 0.598 mA, the corresponding value of x was calculated to be −10.664. Accordingly, the Pb^{2+} concentration was determined to be $10^{-10.664}$ M, which corresponds to 4.4987×10^{-3} ppb.

$$\Delta I_d = 0.598mA = y$$

$$x = -10.664$$

Considering the sample pretreatment process, 57 μL of 1 M NaOH was first added to 5 mL of the original SLRS-6 solution for pH adjustment. The sample was then subjected to conductivity calibration followed by a 4-fold dilution, and an additional 10-fold dilution during measurement. Therefore, the original Pb^{2+} concentration was back-calculated as follows:

$$\text{Original concentration} = 4.4987 \times 10^{-3}\ \text{ppb} \times 5057/5000 \times 4 \times 10$$

$$\text{Original concentration} = 0.182\ \text{ppb}$$

References

(1) Rice, S. A. *Diffusion-limited reactions*; Elsevier, 1985.

(2) Smoluchowski, M. v. Versuch einer mathematischen Theorie der Koagulationskinetik kolloider Lösungen. *Zeitschrift für physikalische Chemie* **1918**, *92* (1), 129-168.

(3) Schroeder, D. V. *An introduction to thermal physics*; Oxford University Press, 2020.

(4) Kholodenko, A. L.; Douglas, J. F. Generalized Stokes-Einstein equation for spherical particle suspensions. *Physical Review E* **1995**, *51* (2), 1081.
